# Short-term dietary changes can result in mucosal and systemic immune depression

**DOI:** 10.1038/s41590-023-01587-x

**Published:** 2023-08-14

**Authors:** Francesco Siracusa, Nicola Schaltenberg, Yogesh Kumar, Till R. Lesker, Babett Steglich, Timur Liwinski, Filippo Cortesi, Laura Frommann, Björn-Phillip Diercks, Friedericke Bönisch, Alexander W. Fischer, Pasquale Scognamiglio, Mira J. Pauly, Christian Casar, Yotam Cohen, Penelope Pelczar, Theodora Agalioti, Flemming Delfs, Anna Worthmann, Ramez Wahib, Bettina Jagemann, Hans-Willi Mittrücker, Oliver Kretz, Andreas H. Guse, Jakob R. Izbicki, Kara G. Lassen, Till Strowig, Michaela Schweizer, Eduardo J. Villablanca, Eran Elinav, Samuel Huber, Joerg Heeren, Nicola Gagliani

**Affiliations:** 1 Department of General, Visceral and Thoracic Surgery, University Medical Center Hamburg-Eppendorf, Hamburg, Germany; 2 Department of Biochemistry and Molecular Cell Biology, University Medical Center Hamburg-Eppendorf, Hamburg, Germany; 3 Department of Microbial Immune Regulation, Helmholtz Centre for Infection Research, Braunschweig, Germany; 4 I. Department of Medicine, University Medical Center Hamburg-Eppendorf, Hamburg, Germany; 5 Systems Immunology Department, Weizmann Institute of Science, Rehovot, Israel; 6 University Psychiatric Clinics, University of Basel, Basel, Switzerland; 7 Bioinformatics Core, University Medical Center Hamburg-Eppendorf, Hamburg, Germany; 8 Core Facility of Electron Microscopy, Center for Molecular Neurobiology ZMNH, University Medical Center Hamburg-Eppendorf, Hamburg, Germany; 9 Institute for Health Service Research, University Medical Center Hamburg-Eppendorf, Hamburg, Germany; 10 Institute for Immunology, University Medical Center Hamburg-Eppendorf, Hamburg, Germany; 11 III. Department of Medicine, University Medical Center Hamburg-Eppendorf, Hamburg, Germany; 12 Immunology, Infectious Diseases and Ophthalmology (I2O) Discovery and Translational Area, Roche Innovation Center, Basel, Switzerland; 13 Centre for Individualised Infection Medicine (CiiM), a joint venture between the Helmholtz-Centre for Infection Research (HZI) and the Hannover Medical School (MHH), Hannover, Germany; 14 Immunology and Allergy Unit, Department of Medicine, Solna, Karolinska Institute and Karolinska University Hospital, Stockholm, Sweden; 15 Division of Microbiome and Cancer, Deutsches Krebsforschungszentrum (DKFZ), Heidelberg, Germany; 16 Hamburg Center for Translational Immunology (HCTI), Hamburg, Germany

**Keywords:** Adaptive immunity, Mucosal immunology, CD4-positive T cells, Infection

## Abstract

Omnivorous animals, including mice and humans, tend to prefer energy-dense nutrients rich in fat over plant-based diets, especially for short periods of time, but the health consequences of this short-term consumption of energy-dense nutrients are unclear. Here, we show that short-term reiterative switching to ‘feast diets’, mimicking our social eating behavior, breaches the potential buffering effect of the intestinal microbiota and reorganizes the immunological architecture of mucosa-associated lymphoid tissues. The first dietary switch was sufficient to induce transient mucosal immune depression and suppress systemic immunity, leading to higher susceptibility to *Salmonella enterica* serovar Typhimurium and *Listeria monocytogenes* infections. The ability to respond to antigenic challenges with a model antigen was also impaired. These observations could be explained by a reduction of CD4^+^ T cell metabolic fitness and cytokine production due to impaired mTOR activity in response to reduced microbial provision of fiber metabolites. Reintroducing dietary fiber rewired T cell metabolism and restored mucosal and systemic CD4^+^ T cell functions and immunity. Finally, dietary intervention with human volunteers confirmed the effect of short-term dietary switches on human CD4^+^ T cell functionality. Therefore, short-term nutritional changes cause a transient depression of mucosal and systemic immunity, creating a window of opportunity for pathogenic infection.

## Abstract (web summary)

Here, the authors show that short-term consumption of energy-dense diets deficient in fiber, similar to eating patterns for many people today, results in a transient depression of the mucosal and systemic immune systems such that susceptibility to bacterial infection is increased.

## Main

A dietary behavior common to omnivorous animals is the consumption of a balanced diet overall, interspersed with the occasional intake of energy-dense food rich in fat. The latter is, however, preferred when the opportunity presents itself^1–6^. In contrast to long-term exposures to hypercaloric diets^7,8^, the health consequences of this short-term consumption of energy-dense nutrients are still unclear. Considering that omnivores have probably undergone an evolutionary pressure to evolve this eating behavior^9–11^, this favors the hypothesis that their biological systems can adapt to reiterated dietary changes, maintaining the host’s homeostasis. However, whether and how the host and its adaptive immune system are able to adapt to short-term alternations between different dietary regimens remains to be tested.

Along the gastrointestinal tract, short-term macronutrient changes can rapidly alter human intestinal microbiota, favoring the growth of certain bacteria over others^12^. However, whether the immune system adapts to changes in the intestinal microbiota as fast as the microbiota does to rapid changes in the diet remains unclear. The intestinal microbiota is the first system that comes into contact with nutrients coming from diets, and it is reasonable to hypothesize that it would be able to absorb potential detrimental changes that short-term dietary interventions could pass on to other biological systems, such as intestinal tissues and immune cells^13^. However, this still needs to be tested.

CD4^+^ T cells are critical mediators of adaptive immunity, and their mitochondrial fitness, as well as their capacity to rewire their own metabolism in response to environmental changes, is crucial to perform effector functions^14–16^. It has been suggested that diurnal patterns of food consumption or consumption of diets rich in sugar have an effect on the predisposition to develop CD4^+^ T cell-mediated intestinal damage or obesity and metabolic syndromes^17,18^. However, it is still unclear whether mucosal and systemic CD4^+^ T cells respond to short-term changes in diets, an eating behavior still typical of modern society. Whether these short-term dietary changes can breach intestinal and extraintestinal immunity also remains to be tested.

Here, we show that every reiterated short-term alternation from diets rich in fiber to fiber-poor feast diets (mimicking our social eating behavior) alters the metabolic, transcriptional and immunological landscape of the gastrointestinal tract. The first dietary switch is sufficient to induce a transient state of mucosal and systemic immune depression leading to increased susceptibility to gut-tropic bacterial infections and impaired antigen-specific immunity to a model antigen. This immune depression is characterized by dysfunctional mucosal and peripheral CD4^+^ T cells with altered metabolic fitness. Switching to feast diets led to microbial changes, resulting in reduced microbial provision of fiber metabolites. Both immune depression and CD4^+^ T cell metabolism could be rewired back to normal by reintroducing dietary fiber, ultimately reestablishing mucosal and systemic immunity. Dietary intervention studies with human volunteers confirmed these effects. Taken together, although short-term consumption of an energy-dense diet has the advantage of providing high energy concentrations to the host, our data show that this comes at the cost of a transient immune depression.

## Results

### Host response to reiterated short-term dietary changes

**Fig. 1:**
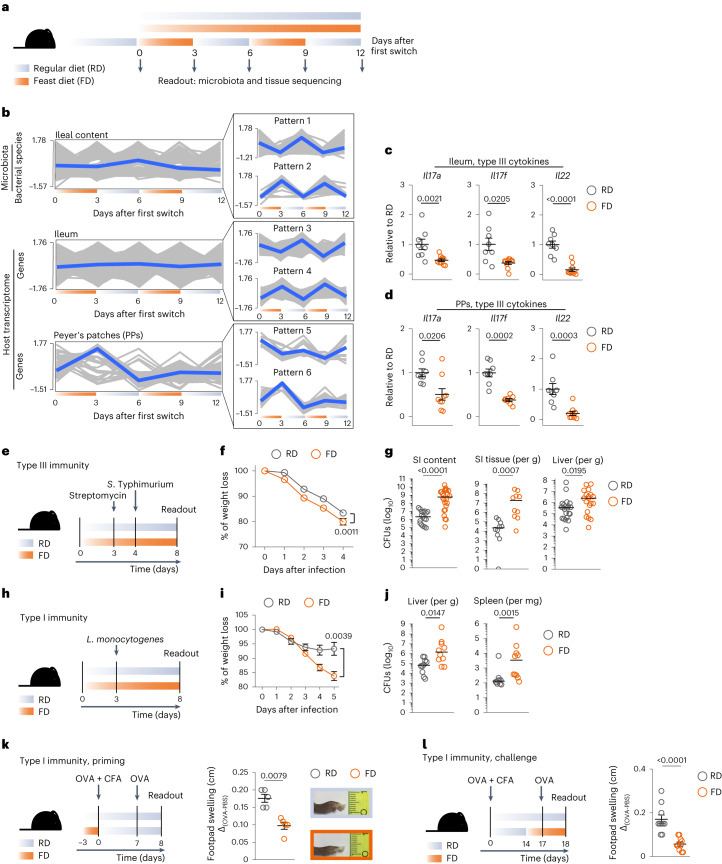
Short-term consumption of FD impairs mucosal and systemic immunity. **a**, Dietary intervention schematic. **b**, Bacterial species (ileal content, top) and genes (ileum and PPs, middle and bottom) significantly changing (*P*_adj_ ≤ 0.05) in at least one time point during dietary intervention. Blue lines represent the average pattern of changes. **c**,**d**, Expression levels of *Il17a*, *Il17f* and *Il22* normalized to *Tbp* and shown as relative to RD, on total ileum (**c**) and PP (**d**) cells. **e**, *Salmonella* Typhimurium infection model. **f**,**g**, Body weight loss (**f**) and CFUs of *S*. Typhimurium in SI luminal content, SI tissue and liver (**g**). **h**, *Listeria monocytogenes* infection model. **i**,**j**, Body weight loss (**i**) and CFUs of *Listeria monocytogenes* in liver and spleen (**j**). **k**,**l**, Left, immunization strategy. Right, swelling of footpad of RD-fed and FD-fed mice during priming (**k**) or challenge (**l**) and representative pictures of swelled-footpads. Data in **b**, top, are one experiment with three to ten mice per group. Data in **b**, middle and bottom, are one experiment with three mice per group. Data in **c** and **d** are a pool of two experiments (ileum, *n* = 8 or 10; PPs, *n* = 8 or 9). Data in **f** are from one experiment (*n* = 12 each), representative of six experiments. Data in **g** are a pool of two experiments (SI content, *n* = 17 or 24, representative of six experiments; SI tissue, *n* = 10 each, representative of four experiments; liver, *n* = 22 or 19, representative of six experiments). Data in **i** and **j** are a pool of two experiments (*n* = 10 each). Data in **k** are from one experiment (*n* = 5 each), representative of three experiments. Data in **l** are a pool of two experiments (*n* = 10 each). Data are shown as mean ± s.e.m. or median (**g**,**j**). *P* values have been determined by two-tailed Wald test (**b**) or two-tailed nonparametric Mann–Whitney *U*-test (**c**,**d**,**f**,**g**,**i**–**l**). Source data

**Extended Data Fig. 1:**
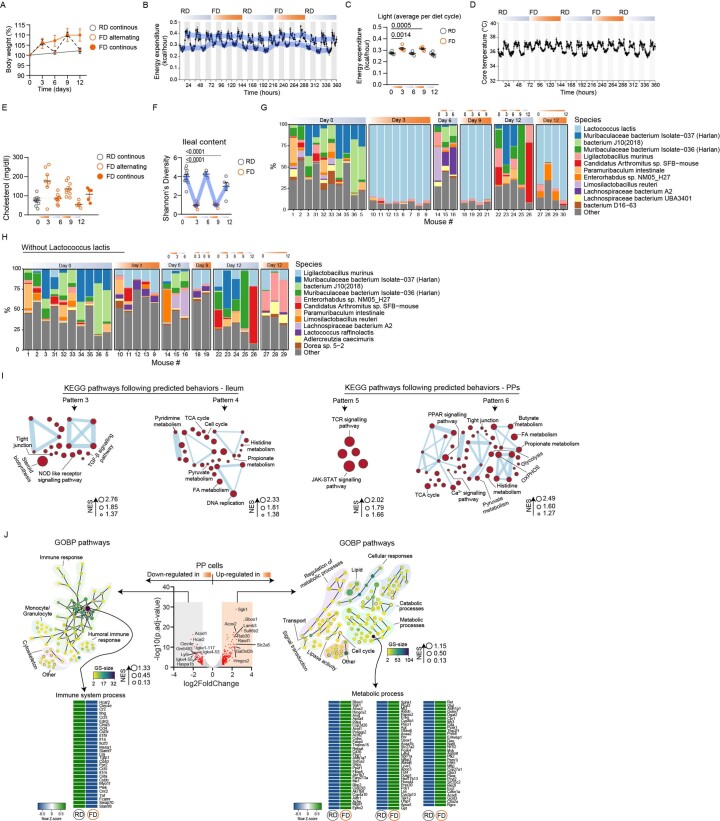
Effects of reiterated short-term dietary changes. (**a**) Body weight gain over dietary intervention. (**b–d**) Energy expenditure (kcal/hour, B), mean of energy expenditure per each dietary switch during light cycle (C) or core temperature (D) measured during indirect calorimetric experiment. White bars represent light cycle, grey bars represent dark cycle; blue lines represent the mean of energy expenditure at each cycle. (**e**) Serum levels of cholesterol (mg/dll) over dietary intervention. (**f**) Shannon’s diversity of ileal content over dietary intervention. (**g**, **h**) Abundance of bacterial species of ileal content over dietary intervention with (G) and without (H) *Lactococcus lactis*; top 12 species with highest counts are shown. (**i**) Enrichment map showing KEGG pathways following the depicted patterns over dietary intervention, as determined by GSEA on whole transcriptome of ileum (left) and PPs (right) (FDR ≤ 0.25). Each node is a pathway and the line connects pathways that have genes in common. The thickness of the line corresponds to the number of shared genes. (**j**) Volcano plot showing DEGs (p.adj≤0.05 and |log2FC|≥1) in total PP cells isolated from S. Typhimurium-infected mice kept on RD or switched to FD, as determined by RNA-sequencing. Three biological replicates per group were sequenced. Enrichment maps showing GO Biological Process pathways enriched in RD or FD, as determined by functional enrichment analysis on down-regulated (left) or up-regulated (right) genes of total PP cells upon switch to FD and infection with S. Typhimurium (FDR ≤ 0.05 and Edge Cutoff<0.4). Data in (A) are a pool of 3 experiments (n = 8 or 10; plotted is mean + SEM per group). Data in (B-D) are one experiment (n = 5). Data in (E) are a pool of 2 experiments (n = 9, 7 or 5). Data in (F-H) are from one experiment (n = 11, 8, 3, 4 or 5). Data in (I-J) are from one experiment (n = 3). Data are shown as mean ± SEM. P-values were determined using a RM one-way ANOVA test with Bonferroni’s comparisons test (C), one-way ANOVA test with Holm-Sidak’s comparisons test (F) or two-tailed Wald-test (J). Source data

To assess the effect of short-term dietary changes on the immune system, we alternated the diet of mice between regular chow (that is, regular diet, RD) and an energy-dense diet rich in animal-derived fat but poor in fiber (that is, feast diet, FD) at 3-day intervals for a total of four dietary switches (Fig. 1a). Host metabolism and intestinal microbiota were analyzed as references for oscillatory patterns, as they rapidly react to short-term dietary interventions^12,18^. We observed that body weight, serum levels of cholesterol, energy expenditure and core temperature changed in an oscillatory fashion at every dietary switch (Extended Data Fig. 1a–e). Similar observations were made for the ileal microbiota, with bacterial species contracting or expanding at every dietary switch (patterns 1 and 2; Fig. 1b, top, and Extended Data Fig. 1f,g). Like others, we observed an abundance of *Lactococcus lactis* in FD-fed mice, but the overall microbial changes were also confirmed after its removal from the dataset (Extended Data Fig. 1h)^19^. We found that the transcriptome of the ileal tissue also showed an oscillatory pattern. In particular, the differentially expressed genes (DEGs) at any given time point during the dietary intervention followed two dichotomous ‘gain–loss’ patterns (that is, patterns 3 and 4; Fig. 1b, middle). In Peyer’s patches (PPs), the major perturbation occurred after the first switch to FD, with gene expression levels being gradually rescued after the subsequent switches to RD (that is, patterns 5 and 6; Fig. 1b, bottom). Potentially random changes in oscillatory patterns were ruled out by permutation tests and by sequencing microbiota and mucosal tissues sampled at 3-day intervals (Supplementary Fig. 1a–e).

Gene set enrichment analysis (GSEA) using the Kyoto Encyclopedia of Genes and Genomes (KEGG) revealed that pattern 3 (decreasing at every switch to FD) of the ileal transcriptome consisted of pathways involved in mucosal homeostasis. Metabolism was the most represented group of pathways for pattern 4 of the ileum (increasing at every FD and decreasing at every RD period) and pattern 6 of PPs (increasing at the first switch to FD, then being gradually rescued after the subsequent switches to RD). Pattern 5 of PPs, decreasing after the first switch to FD, consisted mainly of pathways involved in immune responses, among which the T cell receptor (TCR) and the Janus kinase/signal transducer and activator of transcription signaling pathways had the highest normalized enrichment score (NES) (Extended Data Fig. 1i). In line with this, the type III cytokines *Il17a*, *Il17f* and *Il22*, which are all critical for maintaining intestinal homeostasis and immunity to upcoming pathogenic infections, were significantly downregulated after the first switch to FD (Fig. 1c,d).

These observations depict a dynamic and synchronized response of the different intestinal compartments to reiterated dietary changes. One 3-day switch to FD was sufficient to alter the transcriptomic profiles of immune-related pathways and, in particular, of type III cytokines.

### Short-term consumption of FD impairs immunity

We wondered what pathophysiological consequences a single short-term switch to FD might have on mucosal immunity. We infected mice with *Salmonella enterica* serovar Typhimurium (*S*. Typhimurium), as it invades intestinal tissues mainly through microfold cells in PPs and then disseminates to systemic sites^20,21^ (Fig. 1e). Mice switched to FD lost significantly more weight and had higher numbers of colony-forming units (CFUs) in small intestine (SI) luminal content, SI tissue and liver than control mice left on RD (Fig. 1f,g). In line with this, pathways associated with humoral and cellular immune responses were downregulated in PPs of infected mice switched to FD (Extended Data Fig. 1j).

The consequences of this change in diet also extended to systemic immune responses, as mice switched to FD could not efficiently clear systemic infections caused by *Listeria monocytogenes*, a known inducer of type I immune responses^22^ (Fig. 1h–j). Moreover, mice switched to FD at priming with ovalbumin (OVA) could not efficiently induce footpad swelling when challenged in a classic delayed-type hypersensitivity (DTH) model (Fig. 1k). Similar results were obtained when OVA-primed mice were switched to FD 3 days prior to the induction of a type I DTH reaction (Fig. 1l), showing that a short-term switch to FD during recall responses affected the ability of antigen-experienced CD4^+^ T cells to respond to antigenic challenges.

Taken together, these data show that short-term dietary changes lead to higher susceptibility to mucosal and systemic bacterial infections and impair antigen-specific CD4^+^ T cell immunity to model antigens.

### Short-term consumption of FD affects mucosal CD4^+^ T cells

We aimed to understand the cellular mechanisms predisposing FD-fed mice to a higher susceptibility to *S*. Typhimurium. Considering the type of cytokines modulated by FD (that is, *Il17a*, *Il17f* and *Il22*), we started by characterizing PP CD4^+^ T cells. Indeed, in response to interaction with the intestinal microbiota, CD4^+^ T cells can continuously contribute to mucosal homeostasis via production of type III cytokines^23^, eventually making the host less susceptible to upcoming infections.

**Extended Data Fig. 2:**
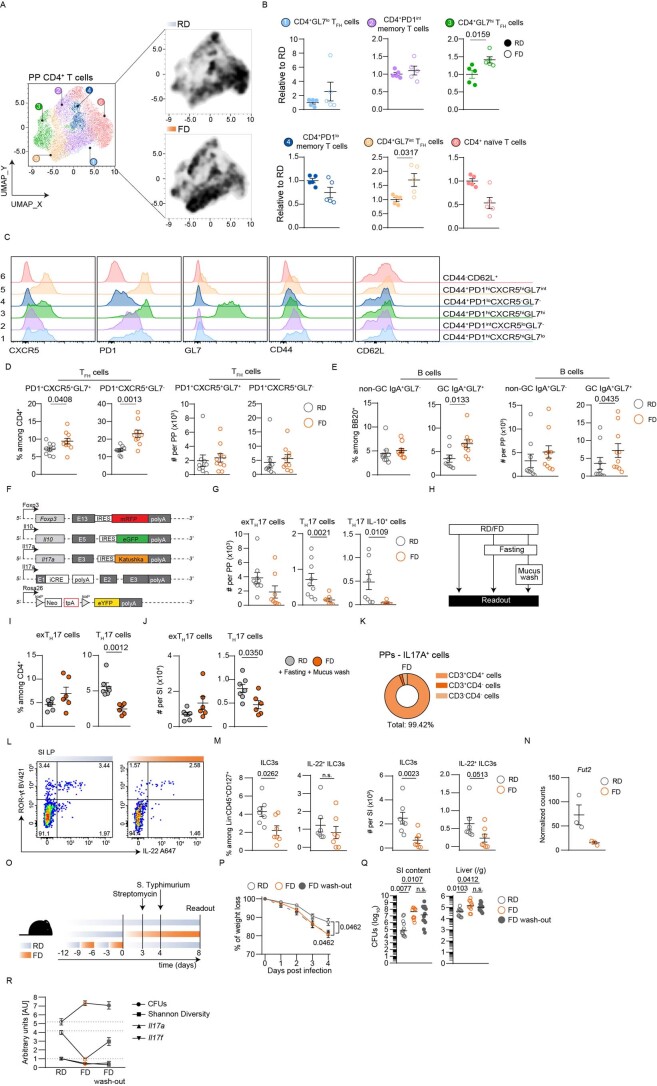
Changes in PPs induced by one 3-day switch to FD. (**a–d**) UMAP (A) and related clusters (B) and parameters (C) calculated on PP CD3^+^CD4^+^ viable T cells isolated from RD- and FD-fed mice. (**d**) Frequencies (left) and numbers (right) of PD1^+^CXCR5^+^GL7^+^ and PD1^+^CXCR5^+^GL7^−^ T_FH_ cells among CD3^+^CD4^+^ T cells in PPs. (**e**) Frequencies (left) and numbers (right) of IgA^+^GL7^−^ (non^−^GC) and IgA^+^GL7^+^ (GC) cells among B220^+^ B cells in PPs. (**f**) Genetic setup of IL-17A fate-mapping reporter mouse. (**g**) Cell numbers per PP of exT_H_17, T_H_17 and IL-10-secreting T_H_17 cells. (**h**) Dietary intervention schematic. (**i–j**) Frequencies (**I**) and numbers (**J**) of SILP exT_H_17 and T_H_17 cells among CD3^+^CD4^+^ T cells, after fasting and mucus wash. (**k**) Pie charts depicting frequencies of IL-17A-producer cells in PPs. (**l**) Representative dot plots of ROR-γt versus IL-22 gated on SILP Lin^-^CD45^+^CD127^+^ viable cells. (**m**) Frequencies (left) and numbers (right) of SILP ILC3s and IL-22-secreing ILC3s. (**n**) Normalized counts of *fut2* gene in ileum cells, as measured by RNA bulk-seq. (**o**) Dietary interventions and S. Typhimurium infection model. (**p, q**) Body weight loss and CFUs of S. Typhimurium in SI luminal content and liver. (**r**) Graph summarizing CFUs in SI luminal content, Shannon’s diversity and il17a/f levels in ileum and PPs as per experimental plan in (O). Data in (A-C) are from one experiment representative of 2 (n = 5 each). Data in (D-E) are a pool of 2 experiments (n = 9 or 10 each). Data in (G) are a pool of 2 experiments, representative of 4 (n = 9, 7, 8 or 5). Data in (I-J) are from 2 experiments (n = 7 or 6). Data in (M) are a pool of 2 experiments (n = 7 each). Data in (N) are from one experiment (n = 3 each). Data in (P) are from one experiment (n = 5 each), representative of 2. Data in (Q) are a pool of 2 experiments (n = 11, 10 or 12). Data are shown as mean ± SEM or median (Q). P.values have been determined by two-tailed non-parametric Mann-Whitney test (B, D-E, G, I-J, M), Brown-Forsythe and Welch ANOVA test (P) or Kruskal-Wallis test with Dunn’s multiple comparisons test (Q). Source data

Based on multiparameter flow cytometry, we identified six different cell clusters (Extended Data Fig. 2a–c) and noticed that while GL7^+^ mature and GL7^−^ T follicular helper (T_FH_) cells were significantly increased upon switching to FD, effector/memory CD4^+^ T cells appeared to be decreased. In line with this, IgA-switched germinal center (GC, B220^+^MHC-II^+^GL7^+^IgA^+^) but not non-GC (B220^+^MHC-II^+^GL7^−^IgA^+^) B cells were also increased (Extended Data Fig. 2d,e). These data show that the adaptive immune system promptly reacts to FD.

**Fig. 2:**
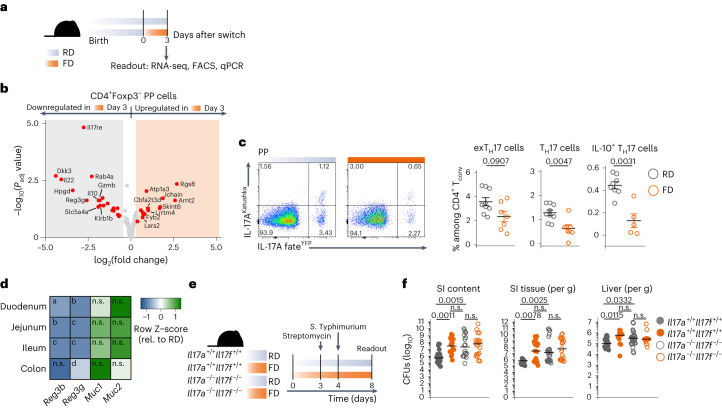
Short-term consumption of FD affects the intestinal CD4^+^ T cell compartment. **a**, Dietary intervention schematic. **b**, Volcano plot showing DEGs (*P*_adj_ ≤ 0.10, |fold change| ≥ 1.3) in FACS-sorted CD4^+^Foxp3^−^ T cells isolated from PPs of RD-fed and FD-fed mice. Two to three biological replicates per group were sequenced. **c**, Left, representative dot plots of exT_H_17 (YFP^+^Katushka^−^) and T_H_17 (YFP^+^Katushka^+^) cells gated on CD3^+^CD4^+^Foxp3^−^ viable conventional T (T_conv_) cells in PPs of RD-fed and FD-fed mice. Right, frequencies of exT_H_17, T_H_17 and IL-10-secreting T_H_17 cells among CD3^+^CD4^+^Foxp3^−^ T_conv_ cells in PPs of RD-fed and FD-fed mice. **d**, Heatmap showing expression level of *Reg3b*, *Reg3g*, *Muc1* and *Muc2* normalized on *Tbp* and shown as relative to RD. **e**, Dietary intervention and *S*. Typhimurium infection model. **f**, CFUs of *S*. Typhimurium in SI luminal content, SI tissue and liver in IL-17A/IL-17F double-knockout and littermate control mice upon switch to FD. Data in **c** are a pool of two experiments (exT_H_17 and T_H_17, *n* = 9 or 7; IL-10^+^ T_H_17, *n* = 8 or 5, representative of four experiments). Data in **d** are a pool of two experiments (*n* = 10 or 9). Data in **f** are a pool of five experiments (SI content, *n* = 23, 16, 18 or 19; SI tissue, *n* = 16, 19, 25 or 19; liver, *n* = 21, 12, 27 or 12). Data are shown as mean ± s.e.m. or median (**f**). *P* values were determined using two-tailed Wald test (**b**), two-tailed nonparametric Mann–Whitney *U*-test (**c**,**d**) or Kruskal–Wallis test with Dunn’s multiple comparison test (**f**). *P* values in **d**: a, 0.0115; b, 0.0005; c, <0.0001; d, 0.0185. Source data

Considering that mice switched to FD had greater difficulty clearing the *S*. Typhimurium infection (Fig. 1f,g), we hypothesized that the impairment of type III mucosal immunity possibly via reduction of effector/memory CD4^+^ T cells and type III cytokines (Fig. 1c–d) was driving the observed phenotype, rather than the expansion of T_FH_ and GC B cells. We therefore focused on CD4^+^ effector T cells and evaluated the transcriptomes of CD4^+^Foxp3^−^ T cells sorted by fluorescence-activated cell sorting (FACS), isolated from PPs of mice switched to FD (Fig. 2a). *Il17re*, *Il22* and *Gzmb*, which all mediate type III mucosal immunity^24–26^, were significantly downregulated (Fig. 2b). Frequencies and numbers of cells with T helper 17 (T_H_17) polarization states were significantly decreased, as were those of T_H_17 cells expressing interleukin-10 (IL-10). A similar trend was also observed for exT_H_17 cells (that is, cells that have expressed IL-17A in the past, but do not currently express it) (Fig. 2c, Extended Data Fig. 2f,g and Supplementary Fig. 2a).

Frequencies and numbers of T_H_17 cells were also significantly reduced in the SI (Extended Data Fig. 2h,j). Of note, although different cellular sources have been described^27,28^, mucosal CD4^+^ T cells were the main producers of IL-17A (Extended Data Fig. 2k), and IL-17F is known to be mostly co-produced^29^.

Collectively, these results indicate that a short-term change in diet alters the immunological landscape of the gastrointestinal tract, resulting in the depression of the intestinal CD4^+^ T cell compartment and in the suppression of the expression of mucosal type III cytokines *Il17a*, *Il17f* and *Il22*.

In line with this, the antimicrobial peptides *Reg3b* and *Reg3g*, known to be induced by IL-17A, IL-17F and IL-22, were significantly downregulated along the SI upon switching to FD (Fig. 2d).

Next, we wondered whether the higher susceptibility of FD-fed mice to *S*. Typhimurium was indeed due to the FD-driven downregulation of IL-17A/IL-17F. Therefore, IL-17A/IL-17F double-knockout mice and littermate controls kept on RD or switched to FD were infected with *S*. Typhimurium (Fig. 2e). RD-fed double-knockout mice were impaired in their ability to control the bacterial infection to the same extent as FD-fed mice. In addition, feeding FD to double-knockout mice did not further increase the bacterial burden (Fig. 2f). These findings indicate that FD-mediated reduction of IL-17A and IL-17F, prior to infection, predisposes the intestine to be more susceptible to *S*. Typhimurium upon switch to FD.

It has been reported that IL-22 derived from type 3 innate lymphoid cells (ILC3s) can protect from *S*. Typhimurium infection by promoting fucosylation of intestinal intraepithelial cells (IECs)^30^. Although reduced overall at the population level, IL-22-secreting ILC3s did not seem to be significantly affected. However, gene expression of ileal *fut2* was downregulated (Extended Data Fig. 2l–n), suggesting that alteration of the fucosylation status of IECs might play an additional role in *S*. Typhimurium susceptibility upon FD consumption.

FD-derived nutrients and reduced bacterial competition due to an altered intestinal microbiota composition could also drive the observed bacterial expansion^31,32^. We therefore infected mice that had been switched back to RD for the last 3 days (that is, the washout period from FD nutrients; Extended Data Fig. 2o). At this time point, all of the tested metabolic parameters (Extended Data Fig. 1a–e) were no longer altered and the overall microbial composition had been restored to the level of RD-fed mice (Extended Data Fig. 1f), while type III cytokines were still downregulated. Mice that underwent this washout period from FD lost a similar amount of weight and had the same increased amount of CFUs as the mice left on FD throughout the whole experiment (Extended Data Fig. 2p–r). These data suggest that FD-derived nutrients and altered microbial composition are not the main drivers of *S*. Typhimurium expansion, supporting the conclusion that the FD-mediated reduction of type III immunity is sufficient to predispose the host for an increased susceptibility to *S*. Typhimurium.

### Short-term consumption of FD impairs antigen-specific CD4^+^ T cells

**Extended Data Fig. 3:**
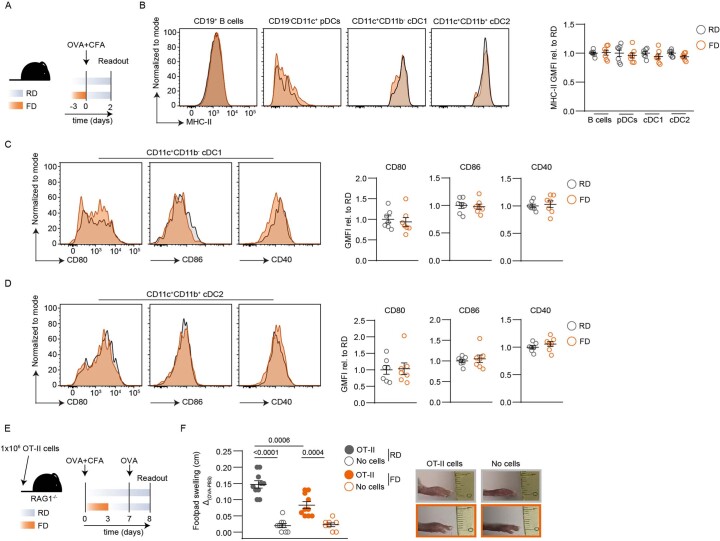
Systemic effects of FD consumption are not mediated by APCs. (**a**) Dietary intervention and immunization strategy. (**b**) Representative histograms (left) and expression level (right) of MHC class II gated on CD19^+^ B, CD11c^+^ pDCs, CD11c^+^CD11b^−^ cDC1 and CD11c^+^CD11b^+^ cDC2 cells isolated from draining LNs of RD- and FD-fed mice. (**c**) Representative histograms (left) and expression level (right) of CD80, CD86 and CD40 gated on CD11c^+^CD11b^−^ cDC1 cells isolated from draining LNs of RD- and FD-fed mice. (**d**) Representative histograms (left) and expression level (right) of CD80, CD86 and CD40 gated on CD11c^+^CD11b^+^ cDC2 cells isolated from draining LNs RD- and FD-fed mice. (**e**) Dietary intervention and immunization strategy. (**f**) (Left) Quantification of footpad swelling of recipient mice that had received 1 × 10^6^ OT-II cells and had been fed RD or FD starting from the day of the adoptive transfer. A group of recipient mice received PBS alone as control. (Right) Representative pictures of swollen footpads of recipient mice. Data in (B-D) are a pool of 2 experiments (n = 7 each). Data in (F) are a pool of 2 experiments (n = 10, 9 or 8). Data are shown as mean ± SEM. P-values were determined using a Brown-Forsythe and Welch ANOVA test with Holm-Sidak’s multiple comparisons test. Source data

Next, we aimed to characterize the cellular mechanisms behind the impairment of systemic immunity and decided to focus on the DTH-OVA model, as this gave us the chance to directly study antigen-specific CD4^+^ T cells. No differences were observed in expression levels of major histocompatibility complex class II (MHC-II), CD80, CD86 and CD40, all critical molecules for antigen presentation and co-stimulation, in B cells, plasmacytoid dendritic cells and conventional dendritic cells in OVA-primed mice upon switch to FD (Extended Data Fig. 3a–d). Footpad swelling could also not be induced in RAG1-knockout mice unless OVA-specific OT-II cells were transferred (Extended Data Fig. 3e,f; RD groups). In contrast, RAG1-knockout recipient mice transferred with OT-II cells that had been switched to FD during priming were not able to mount an efficient DTH reaction to OVA (Extended Data Fig. 3e,f). These findings support the hypothesis that short-term switches to FD directly impact the effector function of CD4^+^ T cells.

**Fig. 3:**
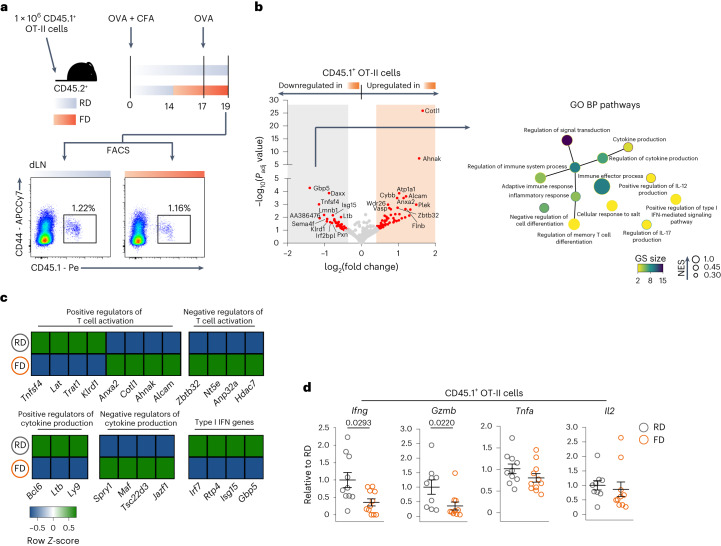
Short-term consumption of FD impairs antigen-specific CD4^+^ T cells. **a**, Top, dietary intervention and immunization strategy. Bottom, representative dot plots of CD45.1^+^ OT-II cells gated on CD3^+^CD4^+^ viable T cells in the draining lymph node (dLN) of RD-fed and FD-fed mice. **b**, Left, volcano plot showing DEGs (*P*_adj_ ≤ 0.10, |fold change| ≥ 1.3) in FACS-sorted CD45.1^+^ OT-II cells isolated from draining lymph nodes of mice kept on RD or switched to FD during systemic OVA challenge. Two samples per group were sequenced, and each sample consisted of a pool of three individual mice from three experiments. Right, enrichment map showing Gene Ontology Biological Process (GO BP) pathways enriched in RD, as determined by functional enrichment analysis on downregulated genes of CD45.1^+^ OT-II cells upon switch to FD (false discovery rate (FDR) ≤ 0.05 and Edge Cutoff < 0.4). GS, gene set. **c**, Heatmap showing expression levels of DEGs selected for having a known function in T cell activation, cytokine production and type I IFN responses in CD45.1^+^ OT-II cells isolated from draining lymph nodes of RD-fed and FD-fed mice during systemic OVA challenge. **d**, Expression levels of *Ifng*, *Gzmb*, *Tnfa* and *Il2* normalized on *Hprt* and shown as relative to RD on FACS-sorted CD45.1^+^ OT-II cells. Data in **d** are a pool of three experiments (*Ifng* and *Tnfa*, *n* = 10 or 11; *Gzmb* and *Il2*, *n* = 9 or 10). Data are shown as mean ± s.e.m. *P* values have been determined by two-tailed Wald test (**b**) or two-tailed nonparametric Mann–Whitney *U*-test (**d**). Source data

In line with this, pathways associated with immune responses were significantly downregulated upon switch to FD in OVA-specific CD4^+^ T cells (Fig. 3a,b). Genes known to negatively regulate T cell activation (*Zbtb32*, *Nt5e*, *Anp32a* and *Hdac7*), as well as negative regulators of cytokine production (*Spry1*, *Maf*, *Tsc22d3* and *Jazf1*), were upregulated upon switch to FD. Conversely, key genes critical for T cell activation (*Tnfsf4*, *Lat*, *Trat1* and *Klrd1*), positive regulators of cytokine production (*Bcl6*, *Ltb* and *Ly9*) and type I interferon (IFN) genes (*Irf7*, *Rtp4*, *Isg15* and *Gbp5*) were downregulated (Fig. 3c). Finally, OVA-specific CD4^+^ T cells exhibited significantly lower levels of *Ifng* and *Gzmb* when challenged with antigen during FD, whereas *Tnfa* and *Il2* showed a decreasing trend (Fig. 3d).

Together, these data show that short-term dietary changes directly impair the effector function of peripheral antigen-specific CD4^+^ T cells.

### Effects of FD are mediated by microbial metabolites

**Fig. 4:**
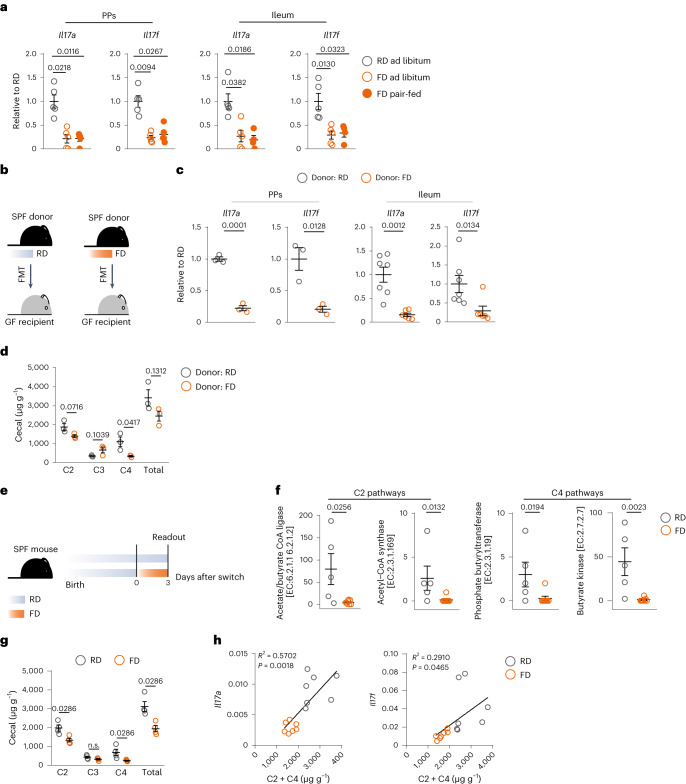
Effects of short-term consumption of FD are mediated by microbial metabolites. **a**, Expression levels of *Il17a* and *Il17f* normalized to *Tbp* and shown as relative to RD, as measured by real-time PCR on total PP (left) and ileum (right) cells isolated from mice kept on RD or switched to FD ad libitum or pair-fed. **b**, Schematic of FMT. SPF, specific-pathogen-free; GF, germ-free. **c**, Expression levels of *Il17a* and *Il17f* normalized to *Tbp* and shown as relative to RD on total PP (left) and ileum (right) cells isolated from germ-free mice that received RD or FD FMT. **d**, Concentration of SCFAs in cecal content of germ-free mice that received RD or FD FMT. **e**, Dietary intervention schematic. **f**, Normalized counts of microbial genes encoding for enzymes involved in C2 and C4 metabolism in ileal contents of RD-fed and FD-fed specific-pathogen-free mice. **g**, Concentration of SCFAs in cecal content of RD-fed and FD-fed specific-pathogen-free mice. **h**, Pearson correlation (two-tailed) of *Il17a* and *Il17f* with C2 and C4 concentrations. Data in **a** are from one experiment (ileum, *n* = 5 or 4; PPs, *n* = 5 each). Data in **c**, left, are from one experiment (*n* = 3 each), representative of two experiments; data in **c**, right, are a pool of two experiments (*n* = 7 or 6). Data in **d** are from one experiment (*n* = 3 each). Data in **f** are from one experiment (*n* = 5 or 8). Data in **g** are from one experiment (*n* = 4 each). Data in **h** are from the same experiments as **d** and **g** (*n* = 7 each). Data are shown as mean ± s.e.m. *P* values have been determined by two-tailed unpaired *t*-test (**c**, left, and **d**), two-tailed *t*-test (**h**), two-tailed nonparametric Mann–Whitney *U*-test (**c**, right, **f** and **g**) or Kruskal–Wallis test with Dunn’s multiple comparison test (**a**). Source data

**Extended Data Fig. 4:**
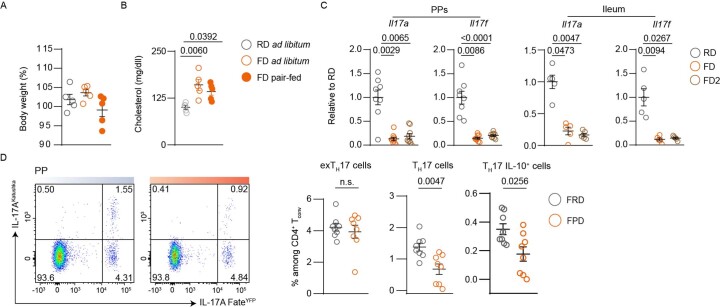
Effects of FD are not dependent on fat and calorie-intake. (**a**) Body weight normalized on day 0 of mice fed RD and FD *ad libitum* or pair-fed. (**b**) Serum levels of cholesterol (mg/dll) of mice kept on RD or switched to FD *ad libitum* or pair-fed. (**c**) Expression levels of *Il17a* and *Il17f* normalized to *Tbp* and shown as relative to RD, as measured by RT-PCR on total PP (left) and ileum (right) cells isolated from RD-, FD- and FD2-fed mice. (**d**) Representative dot plots (left) and frequencies (right) of PP T_H_17 cells among T_conv_ cells isolated from mice fed a low-fat, fiber-deprived diet (FPD) or low-fat, 30% inulin-added high-fiber diet (FRD). Data in (A-B) are from one experiment (n = 5 each).Data in (C) are a pool of 2 experiments (PPs, n = 8 or 9) and from one experiment, representative of two (ileum, n = 5 each). Data in (D) are a pool of 2 experiments (n = 8 each). Data are shown as mean ± SEM. P.values have been determined by Kruskal-Wallis test with Dunn’s multiple comparisons test (B-C) or two-tailed non-parametric Mann-Whitney test (D). Source data

To test whether FD-driven effects were due to a higher caloric intake, we fed mice switched to FD the same amount of calories consumed by RD-fed mice (that is, pair-feeding). Pair-fed mice switched to FD did not gain weight and had similar serum levels of cholesterol and similar levels of *Il17a* and *Il17f* to those of mice switched to FD ad libitum (Fig. 4a and Extended Data Fig. 4a,b). The same results were obtained when mice were switched to a different energy-dense diet containing high levels of sugar, low fiber but no added fat (FD2; Extended Data Fig. 4c). Moreover, mice fed low-fat, fiber-deprived diet (fiber content below 0.3%) showed significantly lower frequencies of PP T_H_17 cells compared with those fed composition-matched high-fiber diet (30% inulin; Extended Data Fig. 4d). These data show that the effects of short-term dietary changes were not dependent on fat and not limited to only one type of FD, but extended to other types of energy-dense diets, all poor in fiber.

Next, we wondered how these fiber-poor diets could mediate their effects. Since the intestinal microbiota can break down dietary fiber into its metabolites and rapidly reacts to short-term dietary changes, we wondered whether FD-driven effects were mediated by the intestinal microbiota. We therefore performed experiments in germ-free mice. Germ-free mice that received FD ileal content showed lower levels of *Il17a* and *Il17f* than germ-free mice that received RD content (Fig. 4b,c). These findings indicate that intestinal type III immune depression is dependent on the composition of the intestinal microbiota, which is changed upon short-term dietary switch to FD.

Key bacterial species in mediating immune homeostasis are those that ferment dietary fiber into short-chain fatty acids (SCFAs)^33^, and one of the major differences between FD and RD is the low amount of dietary fiber contained in FD. We indeed found that the concentration of acetate (C2) and butyrate (C4) was lower in the cecum content of germ-free mice that received FD ileal content compared with those that received RD ileal content (Fig. 4d). Similarly, pathways involved in C2 and C4 synthesis were significantly downregulated in the ileal content of specific-pathogen-free mice switched to FD, as revealed by functional profiling via shotgun metagenomics (Fig. 4e,f). Cecal C2 and C4 concentrations were also lower (Fig. 4g). Gene expression levels of intestinal *Il17a* and *Il17f* positively correlated with C2 and C4 concentrations (Fig. 4h).

These data show that FD-driven effects are not mediated by fat or calorie intake, but by the intestinal microbiota. This led us to hypothesize that the decrease in SCFAs might drive FD-mediated impairment of intestinal and systemic immunity by altering a fundamental mechanism that types I and III immune responses have in common, such as CD4^+^ T cell metabolism^15,34^.

### Short-term consumption of FD alters CD4^+^ T cell metabolism

**Fig. 5:**
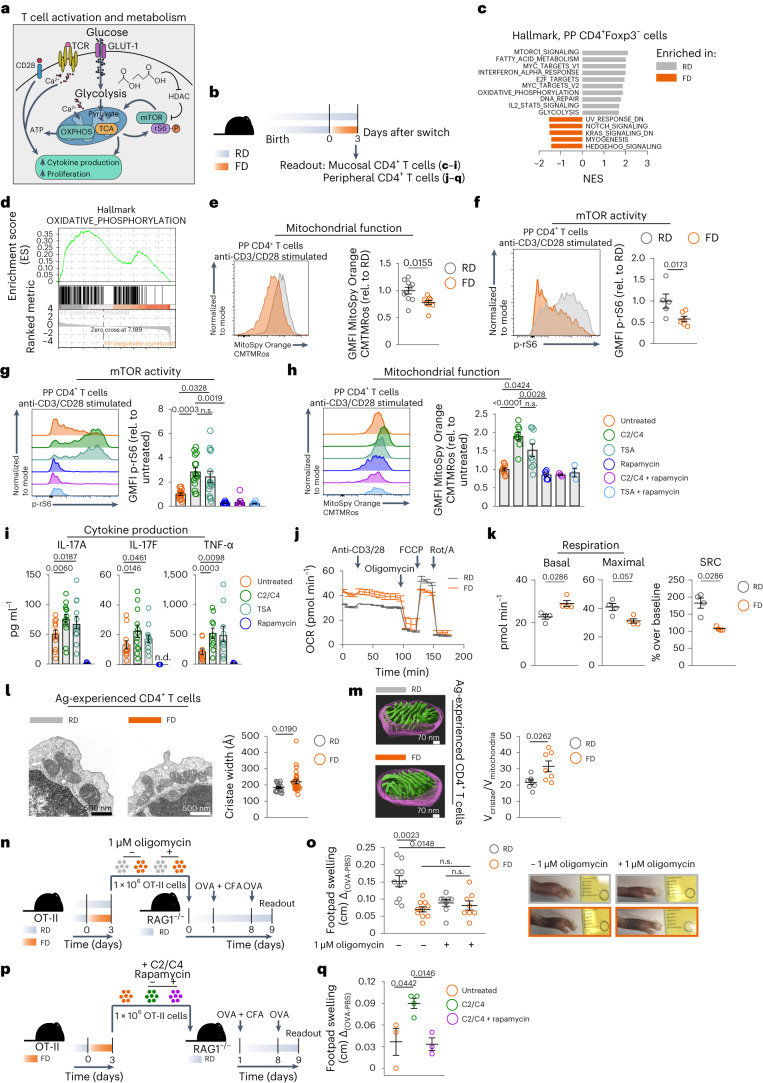
Short-term consumption of FD impairs metabolic fitness of CD4^+^ T cells. **a**, Schematic of T cell metabolism. **b**, Dietary intervention. **c**,**d**, Top ten hallmark pathways (**c**) and OXPHOS (**d**) in PP CD4^+^Foxp3^−^ T_conv_ cells, determined by GSEA (FDR ≤ 0.25). **e–h**, Representative histogram (left) and expression level (right) of MitoSpy Orange CMTMRos (**e**,**h**) or p-rS6 protein (**f**,**g**) in CD4^+^ viable T cells after stimulation of PP cells in the presence or absence of the indicated compounds (**g**,**h**). PP cells in **g** and **h** were isolated from FD-fed mice. GMFI, geometric mean fluorescence intensity; rel., relative. **i**, IL-17A, IL-17F and TNF-α in supernatants of stimulated PP cells with or without the indicated compounds isolated from FD-fed mice. **j**,**k**, Oxygen consumption rate (OCR) (**j**) or basal, maximal respiration and spare respiratory capacity (SRC) (**k**) of splenic CD3^+^CD4^+^ T cells. **l**,**m**, Left, representative TEM images (**l**) or 3D tomography (**m**) of mitochondria from FACS-sorted splenic antigen (Ag)-experienced CD4^+^ T cells. Right, quantification of cristae width (**l**, *n* = 19 or 28; Fiji/ImageJ; scale bar, 500 nm) or volume occupied by cristae within 300 nm of reconstructed mitochondrion (**m**, *n* = 7 each; Etomo; scale bar, 70 nm). **n**, Experimental setup. **o**, Quantification of footpad swelling (left) and representative pictures of swollen footpads (right). **p**, Experimental setup. **q**, Quantification of footpad swelling. Data in **e** are a pool of four experiments (*n* = 10 or 8). Data in **f** are a pool of two experiments (*n* = 5 or 6). Data in **g** are a pool of three (rapamycin groups) or five experiments (*n* = 15 or 9). Data in **h** are a pool of two (rapamycin groups) or five experiments (*n* = 9, 5 or 3). Data in **l** are a pool of four (TNF-α) or five (IL-17A and IL-17F) experiments (*n* = 12, 3, 10 or 4). Data in **j** are from one experiment, representative of two (*n* = 2). Data in **k** are a pool of two experiments (*n* = 4). Data in **o** are a pool of two experiments (*n* = 10 or 8). Data in **q** are from one experiment, representative of two (*n* = 3 or 4). Data are shown as mean ± s.e.m. *P* values have been determined by two-tailed nonparametric Mann–Whitney *U*-test (**e**,**f**,**k**–**m**), one-tailed mixed-effect analysis with Greenhouse–Geisser correction and Sidak’s multiple comparison test (**g**–**i**), Brown–Forsythe and Welch’s analysis of variance (ANOVA) test (**o**) or Kruskal–Wallis test with Dunn’s multiple comparison test (**q**). Source data

To test whether short-term consumption of FD altered the metabolism of both mucosal and peripheral CD4^+^ T cells, we isolated cells from the PPs and spleen of mice switched to FD and evaluated their metabolic fitness (Fig. 5a,b).

PP CD4^+^ T cells exhibited significant downregulation of oxidative phosphorylation (OXPHOS), mammalian target of rapamycin (mTOR) and glycolysis pathways upon switch to FD (Fig. 5c,d). Since metabolism and ability of CD4^+^ T cells to secrete effector cytokines are strictly linked to TCR stimulation^35^, we activated PP CD4^+^ T cells in vitro with anti-CD3/anti-CD28. As a result, PP CD4^+^ T cells of mice switched to FD exhibited lower mitochondrial fitness upon TCR triggering, as evidenced by significantly lower mean fluorescence intensity of MitoSpy Orange CMTMRos, a measure of mitochondrial membrane potential (Fig. 5e). Upon TCR stimulation, the downstream mTOR pathway was also affected by the switch to FD, as shown by significantly lower expression of phosphorylated ribosomal protein S6 (p-rS6) (Fig. 5f), a key target of the mTOR pathway^36,37^.

**Extended Data Fig. 5:**
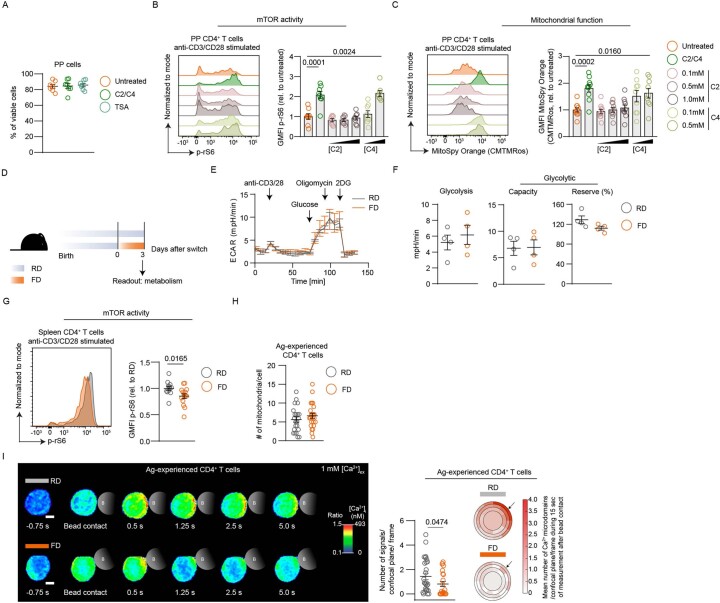
Effects of short-term consumption of FD on CD4^+^ T cell metabolism. (**a**) Viability of PP cells isolated from FD-fed mice after 72 hours of stimulation in the presence or absence of C2/C4 or TSA. (**b, c**) Representative histograms (left) and expression level (right) of p-rS6 protein (B) or Mytospy Orange (CMTMRos) (C) gated on TCRVβ^+^CD4^+^ viable T cells after 72 hours of stimulation of PP cells isolated from FD-fed mice in the presence or absence of the indicated compounds. (**d**) Dietary intervention schematic. (**e, f**) ECAR (E) or glycolysis and glycolytic capacity and reserve (% over baseline, F) measured in CD3^+^CD4^+^ T cells isolated from spleens of RD- and FD-fed mice. (**g**) Representative histogram (left) and expression level (right) of p-rS6 protein gated on TCRVβ^+^CD4^+^ viable T cells after 16 hours of stimulation of total splenocytes. (**h**) Number of mitochondria per cell of FACS-sorted ag-experienced CD4^+^ T cells (n = 22 or 24). Each dot represents the number of mitochondria per cell. (**i**) Representative pictures and quantification of Ca^2+^ microdomains in ag-experienced CD4^+^ T cells from RD- and FD-fed mice after stimulation with beads (‘B’) coated with anti-CD3 and anti-CD28 antibodies. Data in (A) are a pool of 3 experiments (n = 7 each). Data in (B) are a pool of 3 experiments (n = 8 or 7). Data in (C) are a pool of 4 experiments (n = 10 or 9). Data in (E) are from one experiment representative of 2 (n = 2 each). Data in (F) are a pool of 2 experiments (n = 4 each). Data in (G) are a pool of 5 experiments (n = 13 or 15). Data in (I) are a pool of 7 experiments (n = 7 each; RD n = 30 cells and FD n = 22 cells). Data are shown as mean ± SEM. P.values have been determined by two-tailed non-parametric Mann-Whitney (G, I) or one-tailed mixed-effect analysis with Geisser-Greenhouse correction and Dunnett’s multiple comparisons test (B-C). Source data

Since SCFAs were reduced upon switch to FD and can favor cytokine production by enhancing the mTOR pathway through inhibition of histone deacetylase (HDAC)^34^, we treated FD-conditioned PP CD4^+^ T cells with C2 + C4 or the HDAC inhibitor trichostatin A (TSA). The concentrations of C2 and C4 used did not result in cell death and were within the measured in vivo range (Extended Data Fig. 5a). Both C2 + C4 and TSA administration increased phosphorylation of the rS6 of PP CD4^+^ T cells of mice switched to FD, whereas the addition of rapamycin, an mTOR inhibitor, abrogated their effect (Fig. 5g). C2 + C4 treatment or TSA administration also promoted the mitochondrial fitness of PP CD4^+^ T cells of mice switched to FD (Fig. 5h). Secretion of IL-17A, IL-17F and tumor necrosis factor-α (TNF-α) was also increased (Fig. 5i), and all of these effects were abrogated through the addition of rapamycin (Fig. 5g–i). C4 was sufficient to promote both mTOR activity and mitochondrial fitness (Extended Data Fig. 5b,c). These data show that a short-term switch to FD, which is associated with a rapid reduction of SCFAs, impairs mTOR activity and the mitochondrial function of mucosal CD4^+^ T cells, explaining the reduction of cytokine production.

In line with the results obtained in PP CD4^+^ T cells, peripheral CD4^+^ T cells from mice switched to FD showed a higher baseline oxygen consumption rate than those isolated from mice kept on RD (Fig. 5j,k), while glycolysis was not altered (Extended Data Fig. 5d–f, left). However, upon TCR triggering, CD4^+^ T cells of mice switched to FD showed a strong impairment in their capacity to respond to increasing energetic demands, as measured by lower maximal respiration and spare respiratory capacity, SRC (Fig. 5k). No differences were observed in glycolytic capacity or reserve (Extended Data Fig. 5f, right), suggesting that OXPHOS was the main target of FD-driven altered metabolism in CD4^+^ T cells. In line with this, mTOR activity was affected by the switch to FD (Extended Data Fig. 5g). Antigen-experienced CD4^+^ T cells from mice switched to FD showed looser cristae in their mitochondria compared with those of controls (Fig. 5l,m and Supplementary Videos 1 and 2), a phenomenon associated with less efficient OXPHOS^14^, while numbers of mitochondria per cell did not change (Extended Data Fig. 5h). The generation of initial Ca^2+^ microdomains after stimulation was also affected (Extended Data Fig. 5i).

Finally, in vitro treatment of OT-II cells isolated from RD-fed donors with oligomycin abrogated the footpad swelling upon local OVA challenge, decreasing it to the same levels as in recipients that received OT-II cells isolated from FD-fed donors. Notably, oligomycin treatment had no additional effect on OT-II cells isolated from FD-fed donors (Fig. 5n,o). Furthermore, in vitro treatment of OT-II cells from FD-fed mice with C2 + C4 improved their capacity to induce proper footpad swelling upon adoptive transfer. Rapamycin treatment abrogated this effect (Fig. 5p,q).

All together, these data support the hypothesis that a short-term dietary switch to FD renders intestinal and peripheral CD4^+^ T cells less able to rewire their metabolism, thus failing to meet an appropriate energetic level to respond to TCR-mediated activation.

### Reintroducing RD restores mucosal and systemic immunity

Considering that our initial data show the synchronization and strong connection between dietary behaviors and immune pathways, we hypothesized that reintroducing RD would be sufficient to restore efficient immunity.

**Fig. 6:**
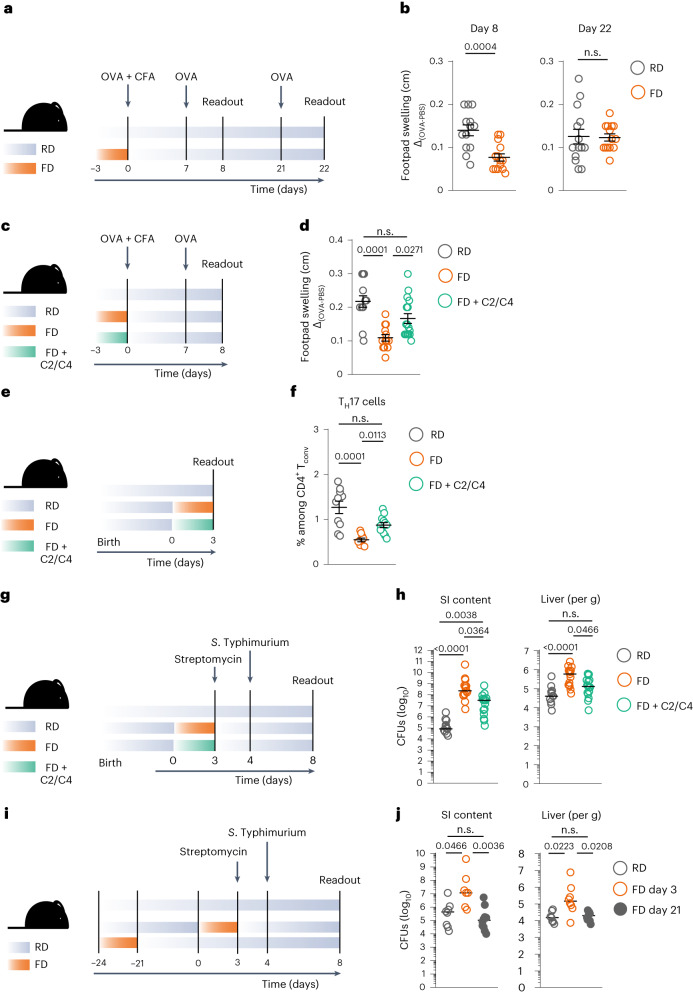
Reintroducing RD restores mucosal and systemic immunity. **a**, Dietary intervention and immunization strategy. **b**, Quantification of footpad swelling of RD-fed and FD-fed mice during priming, 8 or 22 days after priming. **c**, Dietary intervention and immunization strategy. **d**, Quantification of footpad swelling of RD-fed and FD-fed mice (with or without C2 + C4 supplementation) during priming. **e**, Dietary intervention schematic. **f**, Frequencies of T_H_17 cells among CD3^+^CD4^+^ T_conv_ cells in PPs of RD-fed and FD-fed mice (with or without C2 + C4 supplementation). **g**, Dietary intervention and *S*. Typhimurium infection model. **h**, CFUs of *S*. Typhimurium in SI luminal content and liver in RD-fed and FD-fed mice (with or without C2 + C4 supplementation). **i**, Dietary intervention and *S*. Typhimurium infection model. **j**, CFUs of *S*. Typhimurium in SI luminal content and liver in RD-fed mice and mice switched to FD at different time points. Data in **b** are a pool of three experiments (*n* = 13 or 14). Data in **d** are a pool of three experiments (*n* = 13 or 16). Data in **f** are a pool of two experiments (*n* = 10 or 12). Data in **h** are a pool of four experiments (*n* = 13, 15 or 16). Data in **j** are a pool of two experiments (*n* = 7, 8 or 10). Data are shown as mean ± s.e.m. (**b**–**f**) or median (**h**–**j**). *P* values have been determined by two-tailed nonparametric Mann–Whitney *U*-test (**b**) or Kruskal–Wallis test with Dunn’s multiple comparison test (**d**,**f**,**h**,**j**). Source data

**Extended Data Fig. 6:**
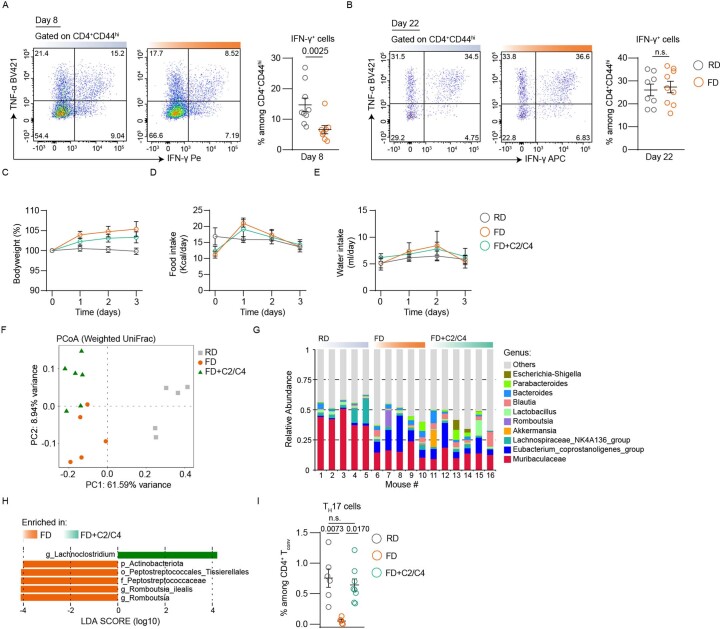
Effects of *in vivo* C2 and C4 supplementation. (**a, b**) Representative dot plots (left) and frequencies (right) of IFN-γ-secreting CD4^+^CD44^hi^ memory T after *in vitro* PMA/Ionomycin re-stimulation isolated from spleens of mice switched to FD or kept on RD, day 8 (A) or 22 (B) after priming. (**c**) Body weight normalized to day 0 of RD- and FD-fed mice (+/− C2/C4 supplementation). (**d**) Food intake (kcal/day) of RD- and FD-fed mice (+/− C2/C4 supplementation). (**e**) Water intake (mL/day) of RD- and FD-fed mice (+/− C2/C4 supplementation). (**f**) PCA of stools isolated from RD- and FD-fed mice (+/− C2/C4 supplementation). (**g**) Relative abundance of bacterial species in stools isolated from RD- and FD-fed mice (+/− C2/C4 supplementation). (**h**) LDA score showing differentially abundant bacteria in FD vs FD + C2/C4 supplementation. (**i**) Frequencies of PP T_H_17 cells among T_conv_ cells isolated from mice fed RD or switched to FD for 3 days with or without C2/C4 supplementation in their drinking water (FD + C2/C4). Data in (A-B) are a pool of 2 experiments (n = 9 or 8). Data in (C-E) are from a pool of 2 experiments (weight gain, n = 9 or 10; food and water, n = 4 or 5, measured per cage). Data in (F-H) are from one experiment (n = 5 or 6). Data in (I) are a pool of 2 experiments (n = 6, 5 or 8). Data are shown as mean ± SEM. P.values have been determined by two-tailed non-parametric Mann-Whitney (A-B) or Kruskal-Wallis test with Dunn’s multiple comparisons test (I). Source data

FD-fed mice were therefore primed with OVA during FD consumption, switched back to RD and challenged 7 or 21 days after the switch to RD (Fig. 6a). Induction of footpad swelling was efficiently restored in mice that had been switched back to RD for at least 21 days, whereas it was still impaired in those that had experienced RD for only 7 days (Fig. 6b). In line with this, IFN-γ-secreting, antigen-experienced CD4^+^ T cells showed no differences in mice that had experienced a longer washout period from FD (that is, 21 days), whereas they were significantly decreased in mice that had experienced RD for only 7 days (Extended Data Fig. 6a,b). These findings show that the capacity to mediate recall responses to antigens that was impaired by a short-term switch to FD could be restored by consuming a fiber-rich diet.

Next, we specifically tested whether direct supplementation of C2 and C4 could improve systemic and mucosal immunity impaired by FD consumption. Although not as efficient as switching back to RD, C2 and C4 supplementation was sufficient to partially rescue the FD-driven systemic immune depression (Fig. 6c,d). Notably, mice switched to FD with C2 and C4 gained as much weight as mice switched to FD only and ate similar amounts of food and drank similar amounts of water (Extended Data Fig. 6c–e). C2 and C4 supplementation could potentially promote the expansion of C4-producing bacteria belonging to the *Lachnoclostridium* genus, indicating a potential positive feedback loop (Extended Data Fig. 6f–h).

In addition, supplementation of FD with C2 and C4 was also sufficient to increase the frequency of PP T_H_17 cells (Fig. 6e,f and Extended Data Fig. 6i) and to ameliorate the susceptibility of FD-fed mice to *S*. Typhimurium (Fig. 6g,h). The still higher, although significantly ameliorated, CFUs in SI content could be explained by known dualistic effects of SCFAs on bacterial growth, including that of *S*. Typhimurium^38,39^. To circumvent this potential caveat, we infected mice switched back to RD with *S*. Typhimurium at different time points after FD consumption. Mice that were fed FD 3 days before infection showed significantly higher CFUs of *S*. Typhimurium than mice kept on RD. However, when mice were infected 21 days after consumption of FD, *S*. Typhimurium infection could be controlled efficiently (Fig. 6i,j).

Taken together, these data show that FD-driven impairment of mucosal and systemic immunity is transient and can be restored by switching back to a fiber-rich diet.

### Short-term consumption of fiber-poor diets in humans

**Fig. 7:**
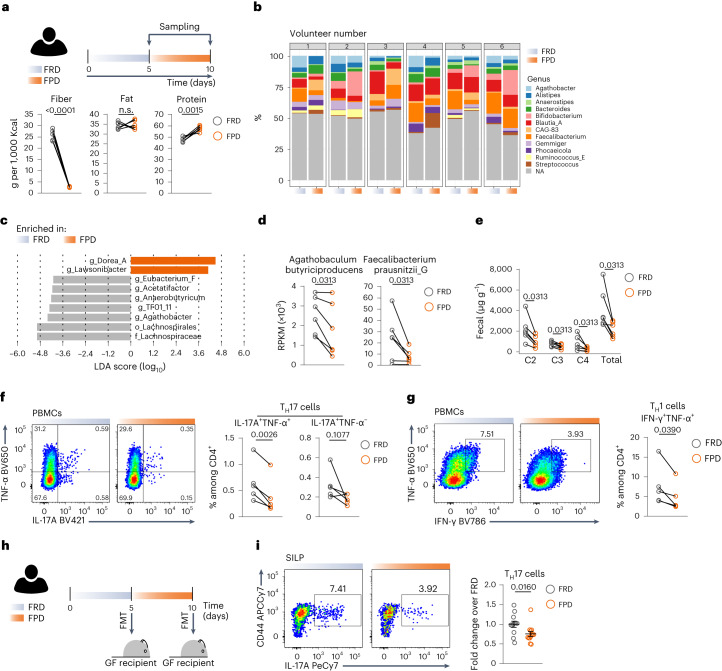
Short-term consumption of low-fiber diet in humans. **a**, Top, human dietary intervention study. Bottom, quantification of fiber, fat and protein intake per volunteer at the two dietary switches. **b**, Microbial composition within each volunteer at the end of each dietary intervention. **c**, Linear discriminant analysis (LDA) score showing differentially abundant bacteria in FRD versus FPD. **d**, Fiber-fermenting, C4-secreting bacterial species shown as reads per kilobase per million mapped reads (RPKM). **e**, Concentration of SCFAs in stools of volunteers before and after each dietary intervention. **f**,**g**, Representative dot plots of human PBMCs gated on viable CD3^+^CD4^+^ cells showing TNF-α versus IL-17A (**f**, left) and TNF-α versus IFN-γ (**g**, left) and frequencies of systemic T_H_17 (**f**, right) and T_H_1 cells (**g**, right) upon switch to FPD. **h**, Schematic showing FMT of FRD-conditioned or FPD-conditioned human stools into germ-free mice. **i**, Representative dot plots of intestinal cells gated on viable TCRβ^+^CD4^+^ cells showing CD44 versus IL-17A (left) and frequencies of intestinal SILP T_H_17 cells upon switch to FPD (right). Data in **a**–**e** are from six different volunteers, and each dot represents one volunteer (*n* = 6). Data in **f** and **g** are from five different volunteers, and each dot represents one volunteer (*n* = 5). Data in **i** are a pool of five experiments (*n* = 12 or 13); each dot represents one germ-free mouse; one to three germ-free mice per donor and per time point were used; a total of four different donors were used. Data are shown as mean ± s.e.m. *P* values were determined using two-tailed paired *t*-tests (**a**,**d**–**g**) or two-tailed nonparametric Mann–Whitney *U*-test (**i**). Source data

**Extended Data Fig. 7:**
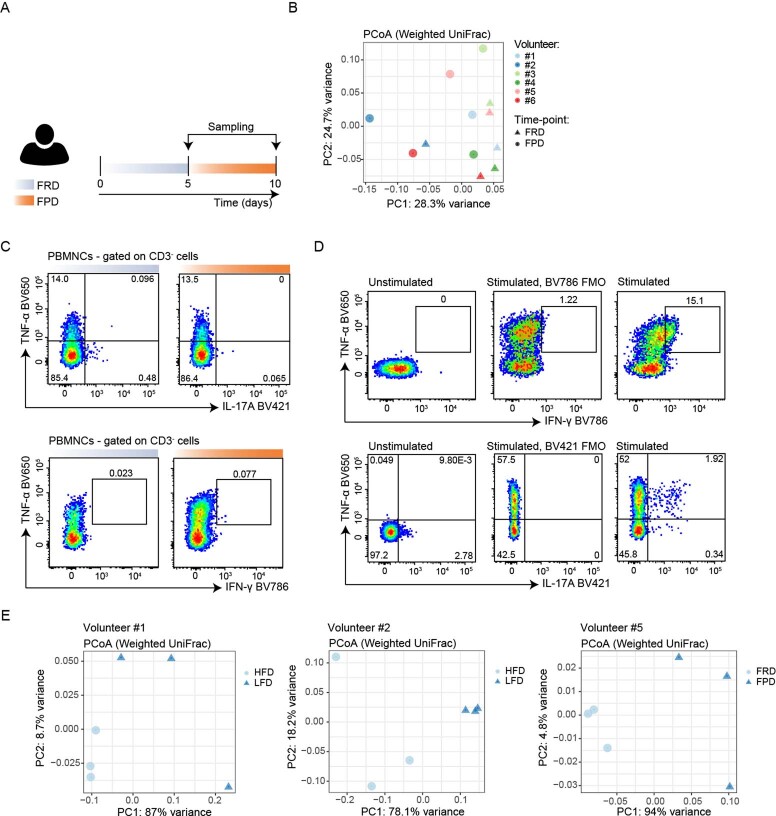
Human dietary intervention study. (**a**) Schematic showing the human dietary intervention study. (**b**) PCA of stools isolated from each volunteer at the end of each dietary interventions. (**c**) Representative dot plots of human PBMCs gated on viable CD3^−^ cells showing specificity of IFN-γ and IL-17A staining. (**d**) Representative dot plots of unstimulated and stimulated (with and without FMOs for anti-IFN-γ-BV786 and anti-IL17A-BV421) human PBMCs gated on viable CD3^+^CD4^+^ cells. (**e**) PCA of stools isolated from GF mice after being colonized with FRD or FPD-human stools. Three volunteers used are shown. Each dot represents one GF mouse; 3 GF mice per volunteer per time-point were used.

Lastly, we performed a human dietary intervention study focused on dietary fiber. Healthy volunteers were offered a fiber-rich diet (FRD) for 5 days and were then switched to a fiber-poor diet (FPD) for an additional 5 days (Fig. 7a). We found that fiber deprivation (that is, switch to FPD) altered the composition of the intestinal microbiota of the volunteers, significantly reducing the abundance of fiber-fermenting bacteria, such as *Eubacterium* and those belonging to the Lachnospiraceae family. Notably, *Agathobaculum butyriciproducens* and *Faecalibacterium prausnitzii*, the main C4 producer in the human gut, were also significantly reduced by the switch to FPD (Fig. 7b–d and Extended Data Fig. 7a,b). Fecal concentrations of SCFAs, including C2 and C4, were significantly decreased by switching from FRD to FPD (Fig. 7e). Furthermore, systemic T_H_17 cells co-expressing IL-17A and TNF-α and T_H_1 cells were significantly decreased in the peripheral blood of the volunteers upon FPD consumption (Fig. 7f,g and Extended Data Fig. 7c,d). Finally, fecal microbial transplantation (FMT) in germ-free mice showed that a fiber-deprived (that is, FPD-conditioned) human microbiota was not able to induce intestinal T_H_17 cells as efficiently as its fiber-rich counterpart (that is, FRD-conditioned) (Fig. 7h,i, Extended Data Fig. 7e and Supplementary Fig. 2b). Taken together, the data show that short-term dietary interventions can significantly alter host responses in both mice and humans.

## Discussion

In this study, we show the synchronization between our dietary behaviors and immune responses and how even a short-term switch from regular to feast diets can have severe effects, causing rapid impairment of intestinal and systemic immunity. This ultimately leads to higher susceptibility to mucosal and systemic bacterial infections and an impaired ability to respond to antigenic challenges with a model antigen. Upon short-term consumption of feast diets, intestinal and systemic CD4^+^ T cells are unable to rewire their metabolism, thus failing to reach an appropriate energetic level to respond to activation. Reintroducing fiber-rich diets efficiently reestablishes T cell metabolism and restores both mucosal and systemic CD4^+^ T cell functions. Finally, the consequences of a short-term switch from high-fiber to low-fiber diets extend to both human and mouse CD4^+^ T cells.

We speculate that in order to guarantee efficient digestion of energy-dense nutrients^6^, a transient downregulation of immunity might have been evolutionarily tolerated. However, this may have come at a price, creating windows of opportunities for pathogenic infections. Higher susceptibility to intestinal infection matches previous findings^31,32,40,41^. Here, we provide evidence for the primary involvement of the immune system, in particular of IL-17A/IL-17F as one of the players in regulating susceptibility to *S*. Typhimurium infection. Our data also suggest a potential connection between FD and the fucosylation status of IECs^30^, but further studies are needed to consolidate this finding.

In addition, our data point toward an overall reduction of the metabolic fitness of mucosal and peripheral CD4^+^ T cells upon short-term dietary changes, resulting in an impaired effector function. Although OVA-specific CD4^+^ T cells were reduced in their capacity to respond to antigenic challenges, whether short-term consumption of energy-dense diets can alter pathogen-specific CD4^+^ T cells in a cognate manner remains to be investigated further.

Along with alterations in the systemic metabolism of mice switched to FD, we found metabolic alterations at the tissue level, with the metabolism of the ileum following an oscillating enrichment–contraction of pathways involved in the tricarboxylic acid (TCA) cycle and metabolism of fatty acids. PPs showed only a transient metabolic hyperactivation mainly induced during the first switch to FD. This raises the question as to how immune cell populations cope with such a state. It has been suggested that CD4^+^ T cells must adapt their metabolism to the environment, undergoing metabolic rewiring in order to survive and function, when homeostasis is perturbed^42,43^. In contrast, mTOR activity and OXPHOS of PP CD4^+^ T cells were decreased upon switch to FD, thus suggesting that CD4^+^ T cells failed to undergo the metabolic rewiring needed to adapt to the PP microenvironment and ultimately to mediate protection.

Consistent with previous works^12,44^, the intestinal microbiota quickly responded to reiterated short-term dietary switches. Bacterial species such as segmented filamentous bacteria (SFB) have been shown to promote the generation of commensal-specific T_H_17 cells^45^, and a decrease in SFB due to high-sugar diets can lead to reduced numbers of these cells^17^. Although SFB showed an initial decrease during our dietary intervention, we show that supplementation of FD with fiber metabolites was sufficient to rescue intestinal T_H_17 cells, bypassing the high amount of sugar of the FD and the absence of SFB. Moreover, fiber-rich human stools efficiently generate intestinal T_H_17 cells when transplanted into germ-free mice. Finally, the absence of SFB alone cannot explain the described systemic immune depression upon switch to FD. Indeed, our results suggest that in addition to the alterations of known T_H_17-inducing bacteria, other mechanisms, such as the reduction in microbially produced fiber metabolites and consequent depression of CD4^+^ T cell metabolic fitness, can explain the decrease in intestinal T_H_17 and systemic T_H_1 cells upon dietary changes.

We propose that the impairment of the mucosal and systemic CD4^+^ T cell compartment is due to a decrease in the mTOR signaling pathway resulting in depression of their mitochondrial fitness probably in response to changes in the fiber-fermenting bacteria leading to a withdrawal of the microbial provision of SCFAs. It has been shown that SCFAs, via their HDAC inhibitory activity^34^, promote the mTOR pathway, which in turn is an orchestrator of mitochondrial function^46–48^. Our data support this finding. SCFAs can also support mTOR activity by working as substrates for TCA^49^, and fatty acid oxidation is important for early T cell activation^50^. Further studies are needed to clarify the role of these co-existing mechanisms during short-term dietary changes.

Finally, our data show that supplementing fiber metabolites could significantly improve types I and III immune responses in the presence of FD, but not to the same extent as by reintroducing diets rich in fiber (that is, RD). This suggests that other dietary components might play an additional role in promoting immunity and might be worth further investigation.

Together, our work uncovers the capacity of short-term dietary changes to orchestrate the dynamic and synchronized behavior of the systemic metabolism, microbiota and immunity, ultimately affecting the host’s health. We ultimately speculate that appropriate diets should be taken into consideration to maximize the efficacy of vaccines and immunotherapies.

## Methods

### Experimental animals, housing conditions and diets

All animal experiments were approved by the Animal Welfare Officers of University Medical Center Hamburg-Eppendorf (UKE) and Behörde für Gesundheit und Verbraucherschutz Hamburg, as well as the Institutional Ethical Committee on Animal Care. C57BL/6J mice were obtained from Charles River Laboratories and Janvier Labs or inbred and raised in UKE animal facilities. All mice were housed at ambient temperature of 20 ± 2 °C, humidity of 55 ± 10% and a light/dark cycle of 12 h/12 h. Additionally, IL-17A/IL-17F double-knockout mice (B6.Cg-Il17a/Il17f^tm1.1Impr^Thy1^a^/J, Charles River Laboratories), cytokine reporter mice (Il17a^Katushka^FoxP3^eRFP^Il10^eGFP^ and Ifng^Katushka^FoxP3^eRFP^Il17a^eGFP^, UKE animal facilities), IL-17A fate-mapping reporter mice (Il17a^CRE^Rosa26eYFP^flx/flx^Il17a^Katushka^FoxP3^eRFP^Il10^eGFP^, UKE animal facilities), RAG1-knockout mice (B6.129S7-Rag1^tm1Mom^/J, Charles River Laboratories) and OT-II mice (B6.Cg-Tg(TcraTcrb)425Cbn/J, UKE animal facilities) bred to express CD45.1 were used. All mice were 10–12 weeks old when experiments were started. Male and female mice were interchangeably used. Mice were randomized before dietary switches, and sample sizes were determined by small pilot experiments. Investigators were not blinded, except for DTH experiments and counting of CFUs. Mice were provided food and water ad libitum, unless stated otherwise. A standard chow diet, referred to as RD (Altromin Spezialfutter, 1328), was used. For dietary intervention, mice received a Western-type diet enriched with cholesterol (referred to as FD) (Research Diets, D14010701), a low-fat, high-sugar diet (referred to as FD2) (Research Diets, D12450B) or composition-matched FPD and FRD (30% inulin added) (ssniff Spezialdiäten, custom made; S5714-E710 and S5714-E716) for 3 days. For reiterated alternations between RD and FD, mice were switched to FD for a 3-day interval for a total of four dietary switches. Control mice were left on RD or FD throughout the intervention period. For pair-feeding experiments, mice switched to FD were fed the exact amount of FD containing the same calories as RD. For fasting experiments, mice were fasted for 4 h prior to being killed. All groups were analyzed on the same day, unless stated otherwise. Germ-free mice received freshly prepared fecal transplantation of RD or FD intestinal content at days 0, 3 and 6 and were analyzed at day 9.

### Indirect calorimetry

Temperature transponders were transplanted into the peritoneum of mice to record body temperature constantly. For indirect calorimetry experiments, mice were single-caged in a thermally and humidity controlled environment using a PhenoMaster (TSE Systems).

### Next-generation sequencing and real-time PCR

PPs were removed from the SI and put in TriFast (Peqlab, 30-2010). The SI was divided into duodenum, jejunum and ileum, and each segment was put in TriFast. Total RNA was purified using a NucleoSpin RNA II Kit (Macherey-Nagel), according to manufacturer’s instructions. RNA sequencing was performed on total PPs, ileum or FACS-sorted CD4^+^Foxp3^−^ or CD45.1^+^ OT-II viable T cells isolated from PPs or draining lymph nodes, respectively.

For library preparation of total PPs and ileum samples, complementary DNA was used as input to construct 250~300 base pair (bp) insert cDNA libraries using a NEBNext Ultra RNA Library Prep Kit (New England Biolabs). Indices were included to multiplex multiple samples. In brief, mRNA was purified from total RNA using poly-T oligo-attached magnetic beads. After fragmentation, the first strand of cDNA was synthesized using random hexamer primers, followed by second-strand cDNA synthesis. For FACS-sorted cells, total RNA was amplified using a SMART-Seq v4 Ultra Low Input RNA Kit for Sequencing (Takara Bio USA) with the double-stranded cDNA being synthesized. The double-stranded cDNA was then purified with AMPure XP beads and quantified with Qubit (Life Technologies). After amplification and purification, the insert size of the library was validated on an Agilent 2100 Bioanalyzer and quantified using quantitative PCR (qPCR). Libraries were sequenced on Illumina NovaSeq 6000 S4 flow cell with PE150. Sequencing quality was assessed with FastQC (v0.11.5), followed by trimming of low-quality bases with Trimmomatic (v0.33) and alignment to the *Mus musculus* genome draft GRCm38.84 using STAR (v2.5.0). Analyses were carried out in R, using Bioconductor packages. Differential expression between conditions was calculated on raw reads using DESeq2. Hierarchical clustering with complete linkage to discover groups of genes showing similar expression patterns was then applied, heuristically cutting the tree to produce six clusters. According to the way DEGs were changing during the dietary oscillations, the six clusters were then grouped into two clusters. cDNA was synthetized using SuperScript III Reverse Transcriptase (Invitrogen) and quantified by TaqMan Gene Expression Assay-based real-time PCR (Thermo Fisher) using the indicated probes on a StepOnePlus system (Applied Biosystems). Expression values of target genes were normalized to values of TATA-box binding protein (*Tbp*) or hypoxanthine guanine phosphoribosyl transferase (*Hprt*) by the change-in-threshold method (2^−ΔCT^). Normalized values were further expressed as fold change over RD. Probes used were *Il17a* (Mm00439618_m1), *Il17f* (Mm00521423_m1), *Il22* (Mm01226722_g1), *Ifng* (Mm01168134_m1), *Reg3b* (Mm00440616_g1), *Reg3g* (Mm00441127_m1), *Muc1* (Mm00449604_m1), *Muc2* (Mm00458310_g1), *Gzmb* (Mm00442837_m1), *Tnfa* (Mm00443258_m1) and *Il2* (Mm00434256_m1).

### Flow cytometry and cell sorting

Single cell suspensions were obtained from PPs, spleens, draining lymph nodes, and SI lamina propria (SILP). In brief, the SI was incubated with dissociation solution at 37 °C for 20 min while shaking to remove epithelial cells (1x HBSS without Ca^2+^ and Mg^2+^ supplemented with 10 mM HEPES, 10% FBS and 0.145 mg ml^−1^ dithiothreitol (DTT)), followed by incubation with digestion solution at 37 °C for 45 min while shaking (RPMI 1640 supplemented with 10% FBS, 0.1 mg ml^−1^ collagenase D, 0.1 mg ml^−1^ DNase I, 1 mM MgCl_2_ and 1 mM CaCl_2_). Percoll gradient (40–80%) was run. For staining, cells were incubated with 10 μg ml^−1^ anti-FcγRII/III (2.4G2, 1:100) in FACS buffer (PBS/0.1% BSA/2 mM EDTA) on ice for 10 min. For surface staining, the following antibodies were used: anti-CD3 (1:200), anti-CD4 (1:400), anti-CD8 (1:400), anti-CD11c (1:200), anti-CD11b (1:200), anti-TCRγδ (1:200), anti-NK1.1 (1:200), anti-B220 (1:400), anti-CXCR5 (1:200), anti-PD1 (1:150), anti-CD69 (1:300), anti-CD44 (1:200), anti-CD62L (1:400), anti-CD45.2 (1:400), anti-CD45.1 (1:300), anti-CD127 (1:150), anti-Gr1 (1:400), anti-CD19 (1:200), anti-FceRa (1:200) and anti-F4/80 (1:200).

For intracellular staining of cytokines, cells were stimulated with 10 ng ml^−1^ phorbol 12-myristate 13-acetate (PMA) and 1 μg ml^−1^ ionomycin in RPMI 1640 supplemented with 10% FBS for 3 or 4 h in the presence of brefeldin A (5 μg ml^−1^) in a cell incubator. For the evaluation of IL-22 production from ILC3s, SILP cells were restimulated in IMDM supplemented with 10% FBS in the presence of 50 ng ml^−1^ recombinant murine IL-23 (rmIL-23) and 100 ng ml^−1^ rmIL-1β. Afterward, cells were stained for surface markers, fixed and permeabilized using a BD Cytofix/Cytoperm Kit (BD Biosciences) or eBioscience Foxp3/Transcription Factor Staining Buffer Set (eBioscience), and stained intracellularly with anti-IL-17A (1:200), anti-TNF (1:600), anti-IFN-γ (1:200), anti-IL-22 (1:50) and anti-RORγt (1:200) at room temperature (20–25 °C) in the dark for 45 or 60 min. For assessing mitochondrial fitness, cells were incubated with 50 nM MitoSpy Orange CMTMRos (BioLegend), according to manufacturer’s instructions. For p-rS6 protein staining, cells were fixed in 4% paraformaldehyde (PFA) at room temperature for 15 min, then washed and fixed in 90% ice-cold methanol for 25 min on ice. Cells were then stained intracellularly with anti-Phospho-S6 Ribosomal Protein (Ser235/236) (1:100) at room temperature in the dark for 1 h. Viability of cells was assessed via Fixable Viability Dye eFluor 506 (eBioscience). For the dimensionality reduction analysis of flow cytometry data, uniform manifold approximation and projection (UMAP) was calculated in FlowJo, on viable CD3^+^CD4^+^ T cells. Samples generated were then analyzed via Cyt^51^ in the MATLAB (vR2016a) environment, and clustering via the expectation-maximization Gaussian mixed (EMGM) model was applied herein. An equal number of events was analyzed. Stained samples were acquired on a BD LSRFortessa (BD Biosciences). For cell sorting, a FACSAria IIIu cell sorter (BD Biosciences) was used. Flow cytometric data were analyzed using FlowJo.

### Bacterial infections

Ten-to-twelve-week-old C57BL/6J or IL-17A/IL-17F double-knockout and littermate control mice were switched to FD 3 days prior to oral streptomycin sulfate salt gavage (20 mg per mouse, *S*. Typhimurium infection only) or infection with *Listeria monocytogenes*. Mice were orally infected with 1 × 10^9^ CFUs of *Salmonella enterica* serovar Typhimurium (SL1344) 24 h after streptomycin treatment or intravenously infected with 5 × 10^3^ CFUs of *Listeria monocytogenes* EGD (bacteria provided by H.-W. Mittrücker, UKE). Mice were killed 4 or 5 days after infection. Mice were kept on FD throughout the whole experiment or switched back to RD at the indicated infection day, whereas control mice received RD (*S*. Typhimurium infection only). In some experiments, mice received a mix of C2 (75 mM) and C4 (75 mM) in their drinking water, and SCFAs were replenished every other day. Bacterial load was determined via serial dilutions of indicated organs on MacConkey or LB (Sigma) agar plates incubated at 37 °C for 24 h. Before plating, SI tissue was incubated at 37 °C for 2 h with gentamycin.

### DTH reactions and OT-II T cell transfer

Ten-to-twelve-week-old C57BL/6J mice were subcutaneously immunized with 250 μg of OVA (Sigma-Aldrich) in Complete Freund’s Adjuvant (CFA) (BD Biosciences). Seven, seventeen or twenty-one days after immunization, mice were challenged with OVA (500 μg in 50 μl of PBS) or PBS in their footpads. After 24 h, footpad swelling was measured using a sliding caliper. Prior to challenge, OVA was allowed to aggregate at 85 °C for 10 min. For adoptive transfer experiments, CD45.2^+^ or RAG1-knockout recipient mice were intravenously injected with 1 × 10^6^ CD45.1^+^ OT-II naïve T cells sorted by magnetic-activated cell sorting (MACS), and 24 h later, subcutaneously immunized with 250 μg of OVA in CFA. Seventeen days after immunization, CD45.2^+^ mice were intraperitoneally challenged with 100 μg of OVA in PBS, and 48 h later, they were killed. Seven days after immunization, RAG1-knockout mice were challenged with OVA or PBS in the footpads, and swelling was measured 24 h later. In some experiments, OT-II cells were treated with 1 μM oligomycin, C2 + C4 (1 mM + 0.5 mM) or 25 nM rapamycin in vitro prior to adoptive transfer.

All mice were switched to FD for 3 days either 3 days before first immunization or before footpad or intraperitoneal challenge. Some mice also received C2 + C4 (75 mM each) supplementation in their drinking water starting with FD 3 days prior to priming. Control mice were kept on RD.

### TCR signaling and Ca^2+^ microdomains

Ten-to-twelve-week-old IL-17A fate-mapping reporter mice were switched to FD for 3 days or kept on RD. Three days after dietary switch, antigen-experienced (CD4^+^CD62L^−^CD44^hi^Foxp3^−^) CD4^+^ T cells were FACS-sorted from spleens. Cells were loaded with Fluo-4 AM (10 µM) and Fura Red (20 µM) at room temperature for 50 min, seeded on coverslips coated with BSA (5 mg ml^−1^) and poly-L-lysine (0.1 mg ml^−1^), and imaged with exposure time of 25 ms (40 frames per s) in 14 bit mode using a Dual-View module (Optical Insights, PerkinElmer) to split the emission wavelengths (filters: excitation, 480/40; beam splitter, 495; emission 1, 542/50; emission 2, 650/57). For the detection of Ca^2+^ microdomains, all pixel intracellular calcium concentration ([Ca^2+^]_i_) values of the microdomain had to be at least Δ[Ca^2+^]_i_ = 112.5 nM higher than the frame-specific mean [Ca^2+^]_i_ of the considered cell. For the comparison of subcellular compartments of tonic Ca^2+^ microdomains, every individual cell was matched onto a circular, dartboard-like template. Based on their spatial coordinates, the identified local Ca^2+^ signals were assigned to the corresponding dartboard compartment, which was normalized according to the size of the cells and the start of the measurement. Finally, the dartboard information for all individual cells was aggregated and evaluated. Stimulation of T cells was performed during imaging with antibody-coated (anti-CD3/anti-CD28) protein G beads (Merck Millipore).

### Transmission electron microscopy (TEM)

FACS-sorted splenic antigen-experienced CD4^+^ T cells were centrifuged at 1,000 × *g* for 10 min in a 1.5-ml microcentrifuge tube. Pellets were fixed with 2.5% wt/vol glutaraldehyde and 3% wt/vol PFA in 0.1 M cacodylate for 1 h. After washing and embedding, pieces were osmicated (1% osmium tetroxide in cacodylate buffer). Sections were dehydrated using ascending ethyl alcohol concentrations, followed by two rinses in propylene oxide. Samples were immersed in a 1:1 mixture of propylene oxide and Epon and finally in neat Epon and polymerized at 60 °C. Semithin sections (0.5 µm) were prepared for light microscopy and mounted on glass slides after staining with 1% toluidine blue. Ultrathin sections (60 nm) were examined in an EM902 (Zeiss). Pictures were taken with a MegaView III digital camera (A. Tröndle).

### Electron tomography

Single tilt electron tomography was performed on mitochondria of FACS-sorted splenic antigen-experienced CD4^+^ T cells with a JEOL JEM-2100Plus electron microscope, with 200 kV acceleration voltage and 300 nm thick plastic embedded sections. Diluted (1:20) 15 nm gold particles (fiducials) were applied on the top and bottom of the copper grid. Tomography was performed at ×20,000 magnification, with a starting angle of approximately −50°, ending angle of approximately 50°, and increment of 1°; tilting and image acquisition was done with a JEOL recorder and charge-coupled device (CCD) camera system (EMSIS). Image size was 5,120 × 3,840, with a pixel size of 0.98 nm px^−1^. Final tomogram generation was performed with Etomo and the IMOD plug-in (https://bio3d.colorado.edu/imod/doc/tomoguide.html#TOP and https://bio3d.colorado.edu/imod/doc/etomoTutorial.html). Gold fiducials were used as markers for reconstruction. Final three-dimensional (3D) reconstruction was performed with Imaris. Three surface masks were created. At every five z-slices, inner cristae membrane contour was manually traced. For the inner mitochondrial volume (matrix and cristae) and intermembrane volume, every 20th z-slice contour was manually traced. All missing z-slices were interpolated by Imaris. Final resolution of the 3D models was the same as the resolution of the z-stack images. The pixel size was 1 nm px^−1^. Image size varied owing to cropping processes in tomography generation with Etomo.

### In vitro T cell cultures

Total PP cells from RD-fed or FD-fed mice were cultured for 3 days with plate-bound anti-CD3 (3 μg ml^−1^) and anti-CD28 (3 μg ml^−1^) in the presence or absence of C2 + C4 (1 mM + 0.5 mM, or indicated varying concentrations of C2 and C4 alone), TSA (10 nM), rapamycin (25 nM), C2 + C4 + rapamycin, or TSA + rapamycin; rmIL-2 (2 ng ml^−1^) was added to the culture. For the evaluation of mitochondrial fitness or p-rS6 protein expression of splenic CD4^+^ T cells, splenocytes from RD-fed or FD-fed mice were cultured for 16 h with plate-bound anti-CD3 (3 μg ml^−1^), anti-CD28 (3 μg ml^−1^) and rmIL-2 (2 ng ml^−1^). After 72 or 16 h of culture, cells were incubated with MitoSpy Orange CMTMRos or processed for p-rS6 protein staining, as described before. The concentration of secreted cytokines was measured in duplicates via LEGENDplex MU Th17 Panel (7-plex) (BioLegend), according to manufacturer’s instructions.

### In vivo SCFA supplementation

Ten-to-twelve-week-old C57BL/6J, cytokine reporter or IL-17A fate-mapping reporter mice were switched to FD for 3 days or kept on RD. A mix of C2 (75 mM) and C4 (75 mM) was administered in the drinking water and replenished every other day.

### Gas chromatography with flame ionization detection (GC–FID)-based analysis of SCFAs

Thirty milligrams of cecal content were extracted in 295.5 μl of ethanol, and isobutyric acid was added as an internal standard. If required, samples were homogenized using a TissueLyser (Qiagen). Samples were centrifuged at 13,000 × *g* for 10 min. Supernatants were mixed with 5 μl of 0.8 M NaOH and then evaporated using a vacuum centrifuge. Residual salts were redissolved in 50 μl of ethyl alcohol and 10 μl of 0.6 M succinic acid. Samples were separated by a gas chromatograph (Hewlett Packard 5890 Series II) equipped with a Nukol Fused Silica Capillary Column (15 m × 0.32 mm × 0.25 μm film thickness) using helium as a carrier gas. Temperature (initial, 70 °C) was raised at 30 °C min^−1^ until reaching 100 °C, and then raised at 6 °C min^−1^ until reaching 190 °C. SCFAs were detected with a flame ionization detector. Ethyl alcohol vials were run between each sample duplicate. Peaks were integrated by comparing retention times and peak areas to standard chromatograms.

### Microbiome sequencing and analysis

Intestinal mucosal and fecal DNA were extracted and purified using a PureLink Microbiome DNA Purification Kit (Invitrogen) or ZymoBIOMICS DNA Microprep Kit, according to manufacturer’s instructions. For shotgun metagenomics sequencing of intestinal mucosal DNA, Illumina libraries were prepared using a Nextera DNA Sample Prep Kit (Illumina, FC-121-1031). Sequencing was carried out on the Illumina NextSeq platform with a read length of 75 bp. Illumina’s bcl2fastq script was applied to generate the fastq files. Quality control was performed using fastp, and reads were aligned to the mm10 mouse genome reference to remove host reads. Remaining reads were mapped against the Genome Taxonomy Database (GTDB, v95) using Kraken 2 (v2.0.8) and Bracken (v2.7) for bacterial species determination. Sparse bacteria present in <10% of samples were removed, samples with <50,000 bacterial reads overall were discarded, and relative abundance of the species was calculated. Curated reads were subsampled using Seqtk (v1.2) and mapped to the UniProt database using DIAMOND (v2.0.15) considering only the top hit and an e-value < 0.0001. All analyses of these experiments were performed in Python.

The DNA library for metagenomics sequencing of human fecal DNA was performed using a NEBNext Ultra II FS DNA Library Prep Kit (New England Biolabs) for Illumina with parameters as follows: 500 ng of input DNA and 37 °C for 5 min for fragmentation; >550-bp DNA fragments for size selection; primers from a NEBNext Multiplex Oligos for Illumina Kit (New England Biolabs) for barcoding. The libraries were sequenced on the Illumina NovaSeq (2 × 150 bp). Raw reads were trimmed for low quality and filtered against the phiX174 and human hg19 genome with BBDuk (https://sourceforge.net/projects/bbmap). For taxonomic species profiling, all libraries were mapped against the Unified Human Gastrointestinal Genome (UHGG) collection (*n* = 4,644) (10.1038/s41587-020-0603-3) using BBMap (https://sourceforge.net/projects/bbmap). Taxa were filtered for low genome coverage (<20%). For normalization, the read counts were divided by genome length in kilobases minus 50 bp. The resulting reads per kilobase (RPK) were counted up and divided by 1,000,000 (per million scaling factor (PMSF)). Transcripts per million (TPM) = RPK/PMFS of each genome bin. Data were summarized as metagenomics operational taxonomic units (OTUs) into biom format and analyzed with phyloseq (10.1371/journal.pone.0061217) and LEfSe (10.1186/gb-2011-12-6-r60).

### Seahorse assays

Splenic CD4^+^ T cells were MACS-sorted with anti-CD4 microbeads (Miltenyi Biotec), according to manufacturer’s instructions, and processed further for Seahorse XF Mito or Glycolysis Stress Test as described elsewhere^52^. In brief, 0.2 × 10^6^ CD4^+^ T cells per well were plated on a poly-D-lysine-coated (50 μg ml^−1^) 96-well plate in XF media pH 7.4 supplemented with 25 mM glucose, 2 mM L-glutamine and 1 mM sodium pyruvate, and incubated at 37 °C for 30–60 min in a non-CO_2_ incubator. Anti-CD3/anti-CD28 beads (2:1 beads:cells; T Cell Activation/Expansion Kit, Miltenyi Biotec) were injected during the assay. Assays were run on an XFe96 Extracellular Flux Analyzer (Agilent).

### Human dietary intervention

The study was conducted at the I. Department of Medicine, UKE, in accordance with the ethical standards of the review board Ethik-Kommission der Ärztekammer Hamburg. Written informed consent was obtained from all participants before study entry (*n* = 6, all female, 28–50 years of age). No compensation was offered. Inclusion criteria were healthy, lean participants aged 18–50. Exclusion criteria were metabolic and autoimmune diseases, familiarity with hypercholesterolemia, pregnancy and/or breastfeeding. All participants were offered one defined FRD and one defined FPD, both consumed for 5 days. Only participants who were accustomed to a dietary fiber load of at least 20 g per day were included in the study. The participants committed themselves to eat exclusively according to previously defined dietary plans and were provided with essential food and cooking instructions for the given meals. Fiber load was >40 g per day for FRD and <5 g per day for FPD. Macronutrients were kept stable in both phases: total energy intake, 1,700–1,800 kcal; carbohydrate intake, 180–210 g total; fat intake, 56–72 g total. Protein intake was 77–82 g per day for FRD and approximately 100 g per day for FPD. Stool and peripheral blood samples were collected at the end of each dietary switch. Human peripheral blood mononuclear cells (PBMCs) were obtained by gradient separation via Ficoll and were restimulated with 10 ng ml^−1^ PMA and 1 μg ml^−1^ ionomycin in RPMI medium containing 10% FCS for a total of 5 h. Brefeldin A (5 μg ml^−1^) was added after the first 2 h. Cells were then processed as previously described and stained intracellularly with anti-CD3 (OKT3, 1:200), anti-CD4 (OKT4, 1:400), anti-TNF (Mab11, 1:400), IFN-γ (4SB3, 1:200) and IL-17A (BL168, 1:150).

### Statistical analysis

Statistical analysis was performed using GraphPad Prism. Normality was tested using Shapiro–Wilk or Kolmogorov–Smirnov tests. Exact tests and *P* values are shown. *P* values > 0.05 were considered not significant (n.s.).

### Reporting summary

Further information on research design is available in the Nature Portfolio Reporting Summary linked to this article.

## Online content

Any methods, additional references, Nature Portfolio reporting summaries, source data, extended data, supplementary information, acknowledgements, peer review information; details of author contributions and competing interests; and statements of data and code availability are available at 10.1038/s41590-023-01587-x.

## Supplementary information

Supplementary Information Supplementary Figs. 1 and 2.

Reporting Summary

Supplemental Video 1 Representative 3D tomography video of 300 nm reconstructed mitochondrion from FACS-sorted antigen-experienced CD4^+^ T cells isolated from spleen of RD-fed mice. Mitochondrial membrane is depicted in magenta, cristae are depicted in green. Scale bar is 50 nm.

Supplemental Video 2 Representative 3D tomography video of 300 nm reconstructed mitochondrion from FACS-sorted antigen-experienced CD4^+^ T cells isolated from spleen of FD-fed mice. Mitochondrial membrane is depicted in magenta, cristae are depicted in green. Scale bar is 50 nm.

## Data Availability

The data generated or analyzed during this study are included in the manuscript and its Supplementary Information files. RNA sequencing, 16S sequencing and shotgun metagenomics are available in the European Nucleotide Archive (ENA) (PRJEB62783 and PRJEB60925), Sequence Read Archive (SRA) (PRJNA951662) and Gene Expression Omnibus (GEO) (GSE229089). Source data are provided with this paper.

## Source data

Source Data Fig. 1 Source data for Fig. 1.
Source Data Fig. 2 Source data for Fig. 2.
Source Data Fig. 3 Source data for Fig. 3.
Source Data Fig. 4 Source data for Fig. 4.
Source Data Fig. 5 Source data for Fig. 5.
Source Data Fig. 6 Source data for Fig. 6.
Source Data Fig. 7 Source data for Fig. 7.
Source Data Extended Data Fig. 1 Source data for Extended Fig. 1.
Source Data Extended Data Fig. 2 Source data for Extended Fig. 2.
Source Data Extended Data Fig. 3 Source data for Extended Fig. 3.
Source Data Extended Data Fig. 4 Source data for Extended Fig. 4.
Source Data Extended Data Fig. 5 Source data for Extended Fig. 5.
Source Data Extended Data Fig. 6 Source data for Extended Fig. 6.
