# Supplementary material for: Short-term dietary changes can result in mucosal and systemic immune depression
Source: Nat Immunol. 2023 Aug 14;24(9):1473–86. doi: 10.1038/s41590-023-01587-x (PMC10457203; doi:10.1038/s41590-023-01587-x)
Supplement: Supplementary file 1 — Supplementary Figs. 1 and 2. [file 41590_2023_1587_MOESM1_ESM.pdf]

nature immunology

Article https://doi.org/10.1038/s41590-023-01587-x

Short-term dietary changes can result in mucosal and systemic immune depression

In the format provided by the authors and unedited

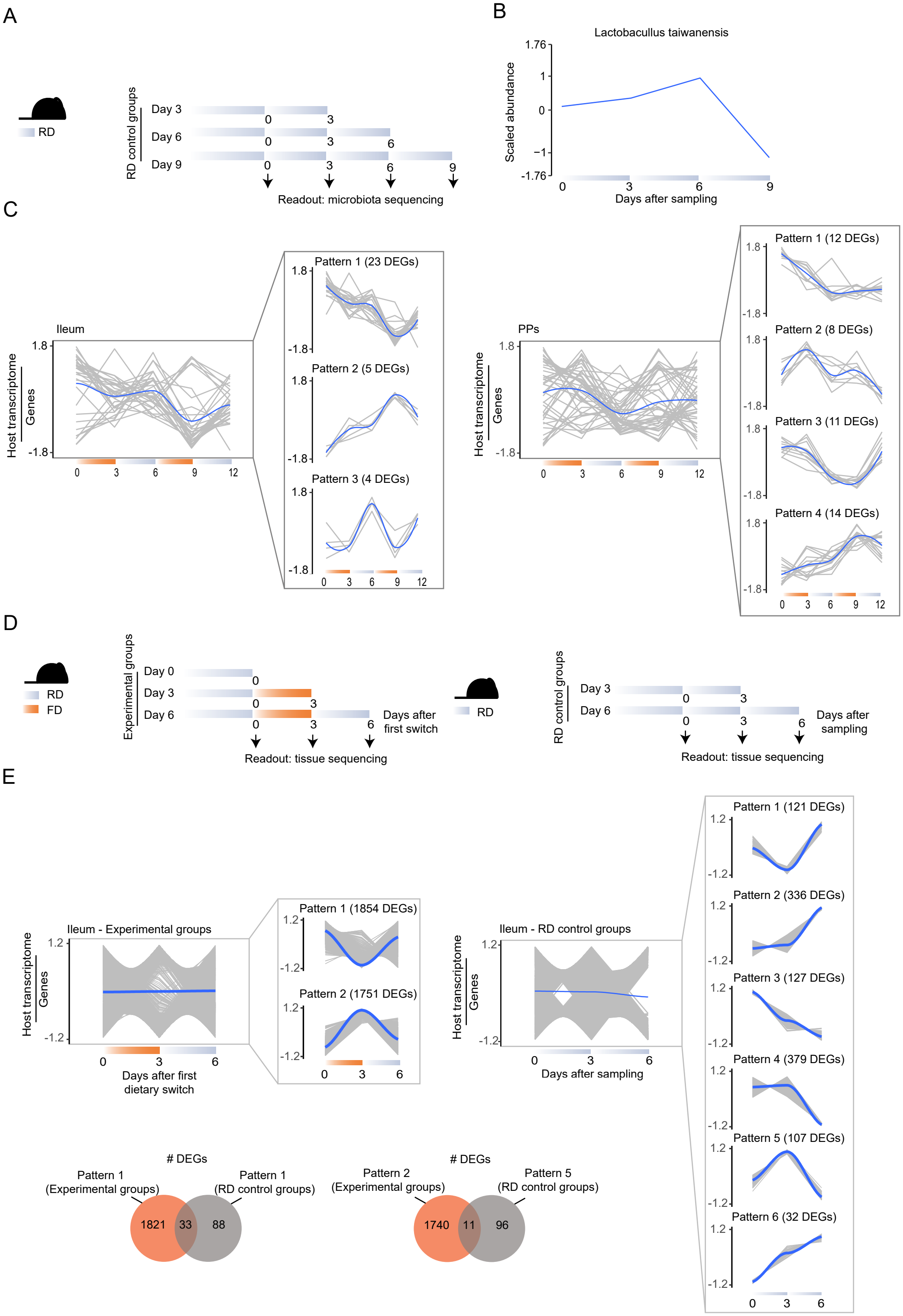

Supplementary Figure 1. Microbial and gene oscillations are not a result of random noise. (A) Experimental setup of dietary intervention. Ileal content was sampled every 3 days from RD-fed mice. (B) Abundance of one bacteria species only (Lactobacullus taiwanensis) is significantly changing, when ileal content of RD-fed mice is sampled at 3 days interval (n=3 each). (C) Representative randomized shuffle (1 of 50 in total) showing changes in gene expression in ileum (left) and PPs (right) of the dataset in Figure 1B after clustering in patterns. Labels of the samples were randomly shuffled (50 randomized shuffles) in order to test whether by chance oscillating patterns could be observed. (D) Schematic showing dietary intervention and control groups of a new dataset. (E) Top. Genes significantly changing (p.adj≤0.05) in all pairwise comparisons of ileum isolated from RD-fed mice at 3 days interval (left) and during dietary intervention (right), n=3 each. Hierarchical clustering with complete linkage to discover groups of genes showing similar expression patterns was applied, identifying 2 main patterns for the experimental groups and 6 for the control groups. Blue lines represent the average pattern of changes. Bottom. Venn diagram showing the amount of DEGs with oscillating patterns in common between control groups and experimental groups.

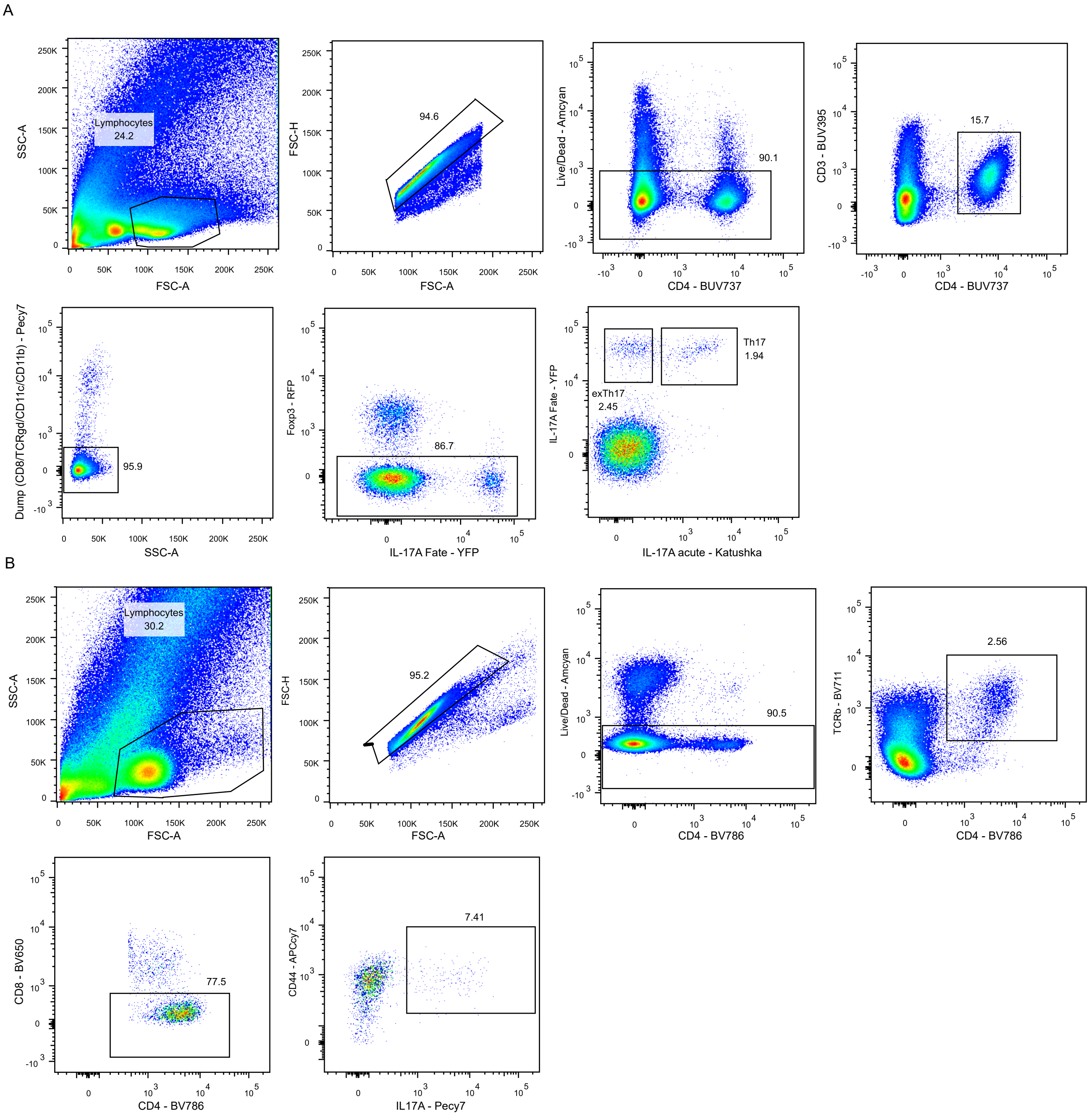

Supplementary Figure 2. Gating strategies. (A) Dot plots exemplifying the gating strategy to detect intestinal exT_{H}17 and T_{H}17 cells in PPs of mice. (B) Dot plots exemplifying the gating strategy to detect intestinal T_{H}17 cells in SI LP of GF mice after FMT with human stools.
