# Supplementary material for: Short-term dietary changes can result in mucosal and systemic immune depression
Source: Nat Immunol. 2023 Aug 14;24(9):1473–86. doi: 10.1038/s41590-023-01587-x (PMC10457203; doi:10.1038/s41590-023-01587-x)
Supplement: Supplementary file 2 — Reporting Summary [file 41590_2023_1587_MOESM2_ESM.pdf]

nature portfolio

Corresponding author(s): Nicola Gagliani
Francesco Siracusa

Last updated by author(s): Jul 8, 2023

Reporting Summary

Nature Portfolio wishes to improve the reproducibility of the work that we publish. This form provides structure for consistency and transparency in reporting. For further information on Nature Portfolio policies, see our Editorial Policies and the Editorial Policy Checklist.

Statistics

For all statistical analyses, confirm that the following items are present in the figure legend, table legend, main text, or Methods section.

n/a	Confirmed	
☐	☒	The exact sample size (*n*) for each experimental group/condition, given as a discrete number and unit of measurement
☐	☒	A statement on whether measurements were taken from distinct samples or whether the same sample was measured repeatedly
☐	☒	The statistical test(s) used AND whether they are one- or two-sided<br>*Only common tests should be described solely by name; describe more complex techniques in the Methods section.*
☒	☐	A description of all covariates tested
☐	☒	A description of any assumptions or corrections, such as tests of normality and adjustment for multiple comparisons
☐	☒	A full description of the statistical parameters including central tendency (e.g. means) or other basic estimates (e.g. regression coefficient) AND variation (e.g. standard deviation) or associated estimates of uncertainty (e.g. confidence intervals)
☐	☒	For null hypothesis testing, the test statistic (e.g. *F*, *t*, *r*) with confidence intervals, effect sizes, degrees of freedom and *P* value noted<br>*Give P values as exact values whenever suitable.*
☒	☐	For Bayesian analysis, information on the choice of priors and Markov chain Monte Carlo settings
☒	☐	For hierarchical and complex designs, identification of the appropriate level for tests and full reporting of outcomes
☐	☒	Estimates of effect sizes (e.g. Cohen's *d*, Pearson's *r*), indicating how they were calculated

Our web collection on statistics for biologists contains articles on many of the points above.

Software and code

Policy information about availability of computer code

Data collection	2100 Bioanalyzer, Illumina NextSeq platform with a read length of 80bp, Illumina NovaSeq 6000 S4 flowcell with PE150, Dual-View module, MegaViewIII digital camera, TSE Phenomaster system, IMARIS, ETOMO, LSR Fortessa and FACS-sort AriaIII machines.
Data analysis	RNA bulk seq. After amplification and purification, insert size of the library was validated on an Agilent 2100 and quantified using quantitative PCR (Q-PCR). Libraries were then sequenced on Illumina NovaSeq 6000 S4 flowcell with PE150 according to results from library quality control and expected data volume. Sequencing quality was assessed with FastQC v. 0.11.5, followed by trimming of low quality bases with Trimmomatic v. 0.33 and alignment to the Mus musculus genome draft GRCm38.84 using STAR v. 2.5.0. All following analysis were carried out in R, using Bioconductor packages. Differential expression between conditions was calculated on raw reads using DESeq2.<br>Ca2+ microdomains. Imaging was carried out with an exposure time of 25 ms (40 frames/s) in 14-bitmode using a Dual-View module (Optical Insights, PerkinElmer Inc.) to split the emission wavelengths (filters: excitation (ex), 480/40; beam splitter (bs), 495; emission 1 (em1) 1, 542/50; em2, 650/57). For the detection of Ca2+ microdomain in cell images, all pixel [Ca2+]i values of the microdomain had to be at least Δ[Ca2+]i = 112.5 nM higher than the frame-specific mean [Ca2+]i of the considered cell.<br>Electron tomography. Tomography was performed at 20.000x mag. and starting angle of approx. -50°, ending angle was at approx. 50°, increment was 1°, tilting and image acquisition with JEOL recorder, CCD camera system by EMSIS. Image size was 5120x3840 with a pixel size of 0,98 nm/px. Final tomogram generation was performed with ETOMO, and the IMOD Plugin v4.11 (https://bio3d.colorado.edu/imod/doc/tomoguide.html#TOP and https://bio3d.colorado.edu/imod/doc/etomoTutorial.html). Gold fiducials were used as marker for reconstruction. Final three-dimensional reconstruction was performed with IMARIS v9.7. Three surface masks were created. Every five Z-Slices inner cristae membrane contour was manually traced. For the inner mitochondrial volume (matrix & cristae) and intermembrane volume every 20th z-slice contour was manually traced. All missing z-slices were interpolated by IMARIS. Final resolution of the 3D models was the same as the resolution of the z-stack images. The pixel size was 1 nm/px, the image size varied due to cropping processes in tomography generation with ETOMO.

Murine microbiome analysis. Sequencing was carried out on the Illumina NextSeq platform with a read length of 75bp. Illumina's bcl2fastq script was applied to generate the fastq files. Quality control was performed using fastp, and the reads subsequently were aligned to the mm10 mouse genome reference to remove all host reads. The remaining reads were than mapped against the Genome Taxonomy Database (GTDB, v95) using kraken2 (v2.0.8) and bracken (v2.7) for bacterial species determination. Sparse bacteria that were present in less than 10% of samples were removed, samples with less than 50K bacterial reads overall were discarded, and relative abundance of the species was calculated for the rest of the samples. Curated reads were subsampled using seqtk (v1.2) and mapped to the uniport database using diamond (v2.0.15) considering only the top hit and an e-value < 0.0001. All analysis of these experiments was performed in python.
Human microbiome analysis. Raw reads were trimmed for low quality and filtered against the phix174 and human hg19 genome with bbduk (sourceforge.net/projects/bbmap). For taxonomic species profiling all libraries were mapped against Unified Human Gastrointestinal Genome collection (n=4,644) (https://doi.org/10.1038/s41587-020-0603-3) using BBMap (sourceforge.net/projects/bbmap/). Taxa were filter for low genome coverage (<20%). For normalization, the read counts were divided by genome length in kilobases minus 50 bp. The resulting reads per kilobase (RPK) were counted up and divided by 1,000,000 (PMSF: per million scaling factor). TPM = RPK/PMFS of each genome bin. Data was summarized as metagenomics OTUs into biom format and analyzed with phyloseq (https://doi.org/10.1371/journal.pone.0061217) and LEfSe (https://doi.org/10.1186/gb-2011-12-6-r60).
UMAP generation from FACS files: Samples generated were then analyzed via Cyt in MatLab (vR2016a) environment, and clustering via Expectation-Maximization Gaussian Mixed (EMGM) model was applied herein.
Cristae quantification: Fiji/Image J.
Flow cytometry. FlowJo v10.
Statistical analysis. GraphPad PRISM 8.

For manuscripts utilizing custom algorithms or software that are central to the research but not yet described in published literature, software must be made available to editors and reviewers. We strongly encourage code deposition in a community repository (e.g. GitHub). See the Nature Portfolio guidelines for submitting code & software for further information.

Data

Policy information about availability of data

All manuscripts must include a data availability statement. This statement should provide the following information, where applicable:

- Accession codes, unique identifiers, or web links for publicly available datasets
- A description of any restrictions on data availability
- For clinical datasets or third party data, please ensure that the statement adheres to our policy

The data generated or analysed during this study are included in the manuscript (and its supplementary information files). All mouse and human data are available on European Nucleotide Archive, ENA (PRJEB62783, PRJEB60925) and Gene Expression Omnibus, GEO (GSE229089).

Human research participants

Policy information about studies involving human research participants and Sex and Gender in Research.

Reporting on sex and gender	For the human intervention study, an open call was published in the internal newsletter of our campus. At the time, only female healthy volunteers responded to the call and were then enrolled.
Population characteristics	Humans enrolled were lean, healthy volunteers from 28 to 50 years of age.
Recruitment	An open call was published in the internal newsletter of our campus. Inclusion criteria were healthy, lean subjects age 18-50. Exclusion criteria were metabolic and autoimmune diseases, familiarity with hypercholesterolemia, pregnancy and/or breast feeding. Subjects were asked to document their usual dietary habits over the course of 3 days and o. nly subjects who were accustomed to a dietary fiber load of at least 20 g/day were included in the study. No compensation was offered.
Ethics oversight	Ethik-Kommission der Ärztekammer Hamburg

Note that full information on the approval of the study protocol must also be provided in the manuscript.

Field-specific reporting

Please select the one below that is the best fit for your research. If you are not sure, read the appropriate sections before making your selection.

☒ Life sciences ☐ Behavioural & social sciences ☐ Ecological, evolutionary & environmental sciences

For a reference copy of the document with all sections, see nature.com/documents/nr-reporting-summary-flat.pdf

Life sciences study design

All studies must disclose on these points even when the disclosure is negative.

Sample size	Sample sizes were based on experience with the described models as well as small pilot experiments. Regarding human data, no sample size was calculated; rather short-chain fatty acids were measured on a small pilot group to confirm that fiber-deprivation was effective.

Data exclusions	No data were excluded from the analysis.
Replication	Replication of experiments are specified in the according figure legends. Data were reproduced and pooled independent experiments or representative data are presented.
Randomization	Mice were randomized before dietary switches by randomly assigning age-matched males and females from different cages/litters to the indicated groups, so that they were equally distributed. Littermate controls were used in all experiments. Human study volunteers were not randomized, as this was not applicable due to the nature of the study itself.
Blinding	In general, investigators were not blinded, as the investigator who planned the experiments, also performed them. Exceptions were DTH experiments and counting of bacterial colonies.

Reporting for specific materials, systems and methods

We require information from authors about some types of materials, experimental systems and methods used in many studies. Here, indicate whether each material, system or method listed is relevant to your study. If you are not sure if a list item applies to your research, read the appropriate section before selecting a response.

Materials & experimental systems

n/a	Involved in the study	
☐	☒	Antibodies
☒	☐	Eukaryotic cell lines
☒	☐	Palaeontology and archaeology
☐	☒	Animals and other organisms
☒	☐	Clinical data
☒	☐	Dual use research of concern

Methods

n/a	Involved in the study	
☒	☐	ChIP-seq
☐	☒	Flow cytometry
☒	☐	MRI-based neuroimaging

Antibodies

Antibodies used

For staining, cells were incubated with 10µg/mL anti-FcγRII/III (BD Biosciences, 553141, 2.4G2) in FACS buffer (PBS/0.1%BSA/2mM EDTA) for 10 minutes on ice. For surface staining, the following antibodies were used: anti-CD3 (BD Biosciences, 569614, 17A2), anti-CD4 (BD Biosciences, 612843, RM4.5 or Biolegend, 100421, GK1.5), anti-CD8 (Biolegend, 100721, 53-6.7), anti-CD11c (Biolegend, 117317, N418), anti-CD11b (Biolegend, 101215, M1/70), anti-TCRγδ (Biolegend, 118123, GL3), anti-NK1.1 (Biolegend, 108713, PK136), anti-B220 (BD Biosciences, 563893, RA3-6B2), anti-CXCR5 (Biolegend, 145505, L138D7), anti-PD1 (Biolegend, 135231, 29F.1A12), anti-CD69 (Biolegend, 104527, H1.2F3), anti-CD44 (Biolegend, 103027, IM-7), anti-CD62L (Biolegend, 104411 or 104431, MEL-14), anti-CD45.2 (Biolegend, 109813, 104), anti-CD45.1 (Biolegend, 110707, A20), anti-CD127 (Biolegend, 135013, A7R34), anti-Gr1(Biolegend, 108405, RB6-8C5), anti-CD19 (Biolegend, 1D3/CD19, 152403), anti-FceRa (Biolegend, 134329, MAR-1), anti-F4/80 (Biolegend, 123119, BM8). For assessing mitochondrial fitness, cells were incubated with 50 nM MitoSpy™ Orange CMTMRos (Biolegend, 424803). For mTOR activity, cells were stained with anti-Phospho-S6 Ribosomal Protein (Ser235/236) (Cell Signalling, 4851, D57.2.2E). For evaluation of cytokine production and/or transcription factor staining, re-stimulated cells were stained intracellularly with anti-IL-17A (Biolegend, 506921, TC11-18H10.1), anti-TNF-α (Biolegend, 506327, MP6-XT22), anti-IFN-γ (Biolegend, 505809, XMG1.2), anti-IL-22 (Biolegend, 516406, Poly5164), anti-RORγt (BD Biosciences, 562894, Q31-378). Viability of cells was assessed via FixableViability Dye eFlour 506 (eBioscience, 65-0866-14). Human cells were stained with: anti-CD3 (BD Bisciences, 750971, OKT3), anti-CD4 (Biolegend, 317407, OKT4), anti-TNFa (Biolegend, 502937, Mab11), IFN-g (Biolegend, 502541, 4SB3) and IL-17A (Biolegend, 512321, BL168). For T cell cultures, purified anti-CD3 (Biolegend, 100339, 145-2C11) and anti-CD28 (Biolegend, 102115, 37.51) antibodies were used.

Validation

anti-FcγRII/III https://www.bdbiosciences.com/en-de/products/reagents/flow-cytometry-reagents/research-reagents/single-color-antibodies-ruo/purified-rat-anti-mouse-cd16-cd32-mouse-bd-fc-block.553141
anti-CD3-BUV395 https://www.bdbiosciences.com/en-de/products/reagents/flow-cytometry-reagents/research-reagents/single-color-antibodies-ruo/buv395-rat-anti-mouse-cd3.569614
anti-CD4-BUV737 https://www.bdbiosciences.com/en-de/products/reagents/flow-cytometry-reagents/research-reagents/single-color-antibodies-ruo/buv737-rat-anti-mouse-cd4.612843
anti-CD4-PeCy7 https://www.biolegend.com/en-us/products/pe-cyanine7-anti-mouse-cd4-antibody-1919
anti-CD8-Pecy7 https://www.biolegend.com/en-us/products/pe-cyanine7-anti-mouse-cd8a-antibody-1906
anti-CD11c- Pecy7 https://www.biolegend.com/en-us/products/pe-cyanine7-anti-mouse-cd11c-antibody-3086
anti-CD11b-Pecy7 https://www.biolegend.com/en-us/products/pe-cyanine7-anti-mouse-human-cd11b-antibody-1921
anti-TCRγδ-Pecy7 https://www.biolegend.com/en-us/products/pe-cyanine7-anti-mouse-tcr-gamma-delta-antibody-7822
anti-NK1.1-Pecy7 https://www.biolegend.com/en-us/products/pe-cyanine7-anti-mouse-nk-1-1-antibody-2840
anti-B220-BV650 https://www.bdbiosciences.com/en-de/products/reagents/flow-cytometry-reagents/research-reagents/single-color-antibodies-ruo/bv650-rat-anti-mouse-cd45r-b220.563893
anti-CXCR5-APC https://www.biolegend.com/en-us/products/apc-anti-mouse-cd185-cxcr5-antibody-8456
anti-PD1-BV711 https://www.biolegend.com/en-us/products/brilliant-violet-711-anti-mouse-cd279-pd-1-antibody-12303
anti-CD69-BV421 https://www.biolegend.com/en-us/products/brilliant-violet-421-anti-mouse-cd69-antibody-7358
anti-CD44-APCcy7 https://www.biolegend.com/en-us/products/apc-cyanine7-anti-mouse-human-cd44-antibody-3933
anti-CD62L-APC https://www.biolegend.com/en-us/products/apc-anti-mouse-cd62l-antibody-381
anti-CD62L-Percp5.5 https://www.biolegend.com/en-us/products/percp-cyanine5-5-anti-mouse-cd62l-antibody-4272
anti-CD45.2-APC https://www.biolegend.com/en-us/products/apc-anti-mouse-cd45-2-antibody-2759

anti-CD45-1-Pe https://www.biolegend.com/en-us/products/pe-anti-mouse-cd45-1-antibody-199
MitoSpy™ Orange CMTMRos https://www.biolegend.com/en-us/products/mitospy-orange-cmtmros-12370
anti-phospho-S6-A647 https://www.cellsignal.com/products/antibody-conjugates/phospho-s6-ribosomal-protein-ser235-236-d57-2-2e-xp-rabbit-mab-alexa-fluor-647-conjugate/4851
anti-IL17A-Pecy7 https://www.biolegend.com/en-us/products/pe-cyanine7-anti-mouse-il-17a-antibody-6013
anti-TNFa-BV421 https://www.biolegend.com/en-us/products/brilliant-violet-421-anti-mouse-tnf-alpha-antibody-7336
anti-IFNG-APC https://www.biolegend.com/en-us/products/apc-anti-mouse-ifn-gamma-antibody-993
anti-IL22-A647 https://www.biolegend.com/en-us/products/alexa-fluor-647-anti-mouse-il-22-antibody-6485
anti-RORγt-BV421 https://www.bdbiosciences.com/en-de/products/reagents/flow-cytometry-reagents/research-reagents/single-color-antibodies-ruo/bv421-mouse-anti-mouse-ror-t.562894
FixableViability Dye eFlour 506 https://www.thermofisher.com/order/catalog/product/65-0866-14?gclid=Cj0KCQjw756lBhDMARIsAEI0AgnzMNXizKM9_qzdc7y92BjBAZYZS8ISt81PIYcqWzUhlbmfR4aVoyAaApM-EALw_wcB&s_kwcid=AL!3652!3!606658601258!e!!g!!fixable%20viability%20dye%20efluor%20506&ef_id=Cj0KCQjw756lBhDMARIsAEI0AgnzMNXizKM9_qzdc7y92BjBAZYZS8ISt81PIYcqWzUhlbmfR4aVoyAaApM-EALw_wcB:G:s&s_kwcid=AL!3652!3!606658601258!e!!g!!fixable%20viability%20dye%20efluor%20506!381166034!75094234991&cid=bid_pca_frg_r01_co_cp1359_pjt0000_bid00000_0se_gaw_bt_pur_con
anti-CD3-BUV737 https://www.bdbiosciences.com/en-de/products/reagents/flow-cytometry-reagents/research-reagents/single-color-antibodies-ruo/buv737-mouse-anti-human-cd3.750971
anti-CD4-FITC https://www.biolegend.com/en-us/products/fitc-anti-human-cd4-antibody-3653
anti-TNFa-BV421 https://www.biolegend.com/en-us/products/brilliant-violet-650-anti-human-tnf-alpha-antibody-7680
anti-IFNg-BV785 https://www.biolegend.com/en-us/products/brilliant-violet-785-anti-human-ifn-gamma-antibody-7986
anti-IL17A BV421 https://www.biolegend.com/en-us/products/brilliant-violet-421-anti-human-il-17a-antibody-7140
anti-CD28 purified https://www.biolegend.com/en-us/products/ultra-leaf-purified-anti-mouse-cd28-antibody-7733
anti-CD3 purified https://www.biolegend.com/en-us/products/ultra-leaf-purified-anti-mouse-cd3epsilon-antibody-7722
anti-CD127- Pecy7 https://www.biolegend.com/en-us/products/pe-cyanine7-anti-mouse-cd127-il-7ralpha-antibody-6192
anti-Gr1-FITC https://www.biolegend.com/en-us/products/fitc-anti-mouse-ly-6g-ly-6c-gr-1-antibody-458
anti-CD19-FITC https://www.biolegend.com/en-us/products/fitc-anti-mouse-cd19-antibody-13615
anti-FceRa-A488 https://www.biolegend.com/de-de/products/alexa-fluor-488-anti-mouse-fcepsilonrialpha-antibody-14525
anti-F4/80-A488 https://www.biolegend.com/de-de/products/alexa-fluor-488-anti-mouse-f4-80-antibody-4073

Animals and other research organisms

Policy information about studies involving animals; ARRIVE guidelines recommended for reporting animal research, and Sex and Gender in Research

Laboratory animals	C57Bl/6J mice were obtained from Charles River and Janvier or in-bred and raised in UKE animal facilities. All mice were housed at ambient temperature of 20±2°C, humidity of 55±10% and a dark/light cycle of 12 hours. Additionally, IL17A/IL17F DKO mice (B6.Cg-Il17a/Il17ftm1.1ImprThy1a/J), cytokine reporter mice (Il17aKatushkaFoxP3eRFPIl10eGFP and IfngKatushkaFoxP3eRFPIl17aeGFP), IL-17A fate-mapping reporter mice (Il17aCRERosa26eYFPflx/flxIl17aKatushkaFoxP3eRFPIl10eGFP), RAG-1 KO (B6.129S7-Rag1tm1Mom/J) and OT-II mice (B6.Cg-Tg(TcraTcrb)425Cbn/J) bred to express CD45.1 were used. All mice were 10-12 weeks old when experiments were started. Male and female mice were interchangeably used and they all were age- and sex-matched.
Wild animals	The study does not involve wild animals.
Reporting on sex	The findings in this study do not apply to only one sex.
Field-collected samples	The study does not report on field-collected samples
Ethics oversight	All animal experiments were approved by the Animal Welfare Officers of University Medical Center Hamburg-Eppendorf (UKE) and Behörde für Gesundheit und Verbraucherschutz Hamburg, as well as the Institutional Ethical Committee on Animal Care

Note that full information on the approval of the study protocol must also be provided in the manuscript.

Flow Cytometry

Plots

Confirm that:

☒ The axis labels state the marker and fluorochrome used (e.g. CD4-FITC).

☒ The axis scales are clearly visible. Include numbers along axes only for bottom left plot of group (a 'group' is an analysis of identical markers).

☒ All plots are contour plots with outliers or pseudocolor plots.

☒ A numerical value for number of cells or percentage (with statistics) is provided.

Methodology

Sample preparation	Single cell suspensions were obtained from PPs, spleen, draining LN (dLN) and SI. After PPs were removed, SI was longitudinally cut, mucus washed away by vigorous shaking in PBS and SI was incubated with dissociation solution at 37°C for 20 minutes while shaking to remove epithelial cells (dissociation solution: 1x HBSS without Ca2+ and Mg2+ supplemented with 10 mM HEPES, 10% FBS and 0.145 mg/mL DTT, Dithiothreitol). SI was then cut into small pieces and incubated with digestion solution at 37°C for 45 minutes while shaking (digestion solution: RPMI-1640 supplemented with 10% FBS, 0.1 mg/

mL Collagenase D, 0.1 mg/mL DNAse I, 1 mM MgCl2 and 1 mM CaCl2). Digested SI was then filtered through a 100µM cell strainer, single cell suspension was resuspended in 40% Percoll solution and stratified on 80% Percoll solution. After density gradient centrifugation, the ring containing lymphocytes was collected and further processed.
Peripheral blood mononuclear cells were obtained after Percoll gradient centrifugation of peripheral blood from healthy volunteers. After gradient centrifugation, the ring containing PBMC was collected and further processed.

Instrument	LSR Fortessa and FACS-sort AriaIII
Software	DB Diva software v8.0.1 and Flowjo v10
Cell population abundance	Frequencies of cell populations are stated in dot plots and graphs.
Gating strategy	In general, lymphocytes were defined using FSC-A vs SSC-A and further gated on singlets using FSC-A vs FSC-H. Dying cells were excluded via the use of cell viability dye. Intestinal CD4+ T cells were typically gated as CD8-TCRgd-CD11c-CD11b- and CD3+/TCRb+CD4+. Further markers were then used to define specific sub-populations as reported in figures and figure legends. CD4+ T follicular helper cells from Peyer's Patches were further defined as CXCR5+PD1+/hiGL7+/-, as reported in figures and figure legends. Germinal center B cells were defined as B220+GL7+IgA+. ILC3s were defined as Lin-CD45+CD127 +RORgt+ cells.

☒ Tick this box to confirm that a figure exemplifying the gating strategy is provided in the Supplementary Information.
